# Bistability, non-ergodicity, and inhibition in pairwise maximum-entropy models

**DOI:** 10.1371/journal.pcbi.1005762

**Published:** 2017-10-02

**Authors:** Vahid Rostami, PierGianLuca Porta Mana, Sonja Grün, Moritz Helias

**Affiliations:** 1 Institute of Neuroscience and Medicine (INM-6) and Institute for Advanced Simulation (IAS-6) and JARA BRAIN Institute I, Jülich Research Centre, Jülich, Germany; 2 Theoretical Systems Neurobiology, RWTH Aachen University, Aachen, Germany; 3 Department of Physics, Faculty 1, RWTH Aachen University, Aachen, Germany; Det Medisinske Fakultet, NTNU, NORWAY

## Abstract

Pairwise maximum-entropy models have been used in neuroscience to predict the activity of neuronal populations, given only the time-averaged correlations of the neuron activities. This paper provides evidence that the pairwise model, applied to experimental recordings, would produce a bimodal distribution for the population-averaged activity, and for some population sizes the second mode would peak at high activities, that experimentally would be equivalent to 90% of the neuron population active within time-windows of few milliseconds. Several problems are connected with this bimodality: 1. The presence of the high-activity mode is unrealistic in view of observed neuronal activity and on neurobiological grounds. 2. Boltzmann learning becomes non-ergodic, hence the pairwise maximum-entropy distribution cannot be found: in fact, Boltzmann learning would produce an incorrect distribution; similarly, common variants of mean-field approximations also produce an incorrect distribution. 3. The Glauber dynamics associated with the model is unrealistically bistable and cannot be used to generate realistic surrogate data. This bimodality problem is first demonstrated for an experimental dataset from 159 neurons in the motor cortex of macaque monkey. Evidence is then provided that this problem affects typical neural recordings of population sizes of a couple of hundreds or more neurons. The cause of the bimodality problem is identified as the inability of standard maximum-entropy distributions with a uniform reference measure to model neuronal inhibition. To eliminate this problem a modified maximum-entropy model is presented, which reflects a basic effect of inhibition in the form of a simple but non-uniform reference measure. This model does not lead to unrealistic bimodalities, can be found with Boltzmann learning, and has an associated Glauber dynamics which incorporates a minimal asymmetric inhibition.

## Author summary

Networks of interacting units are ubiquitous in various fields of biology; e.g. gene regulatory networks, neuronal networks, social structures. If a limited set of observables is accessible, maximum-entropy models provide a way to construct a statistical model for such networks, under particular assumptions. The pairwise maximum-entropy model only uses the first two moments among those observables, and can be interpreted as a network with only pairwise interactions. If correlations are on average positive, we here show that the maximum entropy distribution tends to become bimodal. In the application to neuronal activity this is a problem, because the bimodality is an artefact of the statistical model and not observed in real data. This problem could also affect other fields in biology. We here explain under which conditions bimodality arises and present a solution to the problem by introducing a collective negative feedback, corresponding to a modified maximum-entropy model. This result may point to the existence of a homeostatic mechanism active in the system that is not part of our set of observable units.

## Introduction

Correlated activity between pairs of cells was observed early on in the history of neuroscience [1, 2]. Immediately the question arose whether there is a functional interpretation of this observation [3], and this question is still with us. Hypotheses range from synchronous activation of neurons to bind representations of features into more complex percepts [4–7], to the involvement of correlations in efficiently gating information [8]. Direct experimental evidence for a functional role of correlated activity is the observation that the synchronous pairwise activation of neurons significantly deviates from the uncorrelated case in tight correspondence with behaviour. Such synchronous events have been observed in motor cortex [9, 10] at time points of expected, task-relevant information. In primary visual cortex they appear in relation to saccades (eye movements) [11, 12]. Another argument for the functional relevance of correlations is the robustness of signals represented by synchronous activity against noise [13]. Non-Gaussian distributions of membrane potentials of neurons indeed point towards the synchronized arrival of synaptic events [14, 15]. An opposite view regards correlated activity merely as an unavoidable epiphenomenon of neurons being connected and influencing one another [16]. In the worst case, both these views are partly true, prompting us to find ways to distinguish functionally relevant correlated events from the uninformative background.

In the context of experimental paradigms that perform repeated trials, the co-variability of neurons across trials has been termed “noise correlation”. Recurrent network models are able to reproduce and explain the weak magnitude and wide spread across pairs of second-order [17–23] and higher-order correlations [24, 25]. These simple dynamical models effectively map the statistics of the connectivity to the statistics of the activity. Even though they explain the uninformative part of correlated activity, it is unclear how to use them to distinguish this background from departures thereof.

The separation of the noise- or background correlation from functionally meaningful correlation is in addition hampered by the diverse dimensions of information processing’s not being completely orthogonal. Indeed, correlation transmission may be modulated by changes of firing rate [9]. Theory [26, 27] confirmed this entanglement in the regime of Gaussian fluctuating membrane potentials.

The dynamical-model approaches just outlined pivot on a more or less realistic physical description of the network, with some stochastic features. A complementary approach is also possible, fully pivoting on statistical models. The latter try to predict and characterize neuronal activity without relying on a definite physical network model. Statistical models have two convenient features. First, intuitive statistical working hypotheses usually translate into a *unique* statistical model [28, 29]; this fact streamlines the construction and selection of a such a model. For example, the assumption that first- and second-order correlations recorded in an experiments are sufficient to predict the activity recorded in a new experiment, uniquely selects a truncated Gaussian model [29, 30]. Second, a successful statistical model implicitly restricts the set of possible dynamical physical models of the network: only those reflecting the well-modelled statistical properties are acceptable. Statistical models thus help in modelling the actual physical network structure.

A limit case of this kind of statistical models is obtained by choosing probability distributions having maximum entropy under the constraints of experimentally observed quantities [31, 32; in neuroscience see e.g. 33]. The suitability of such maximum-entropy distributions for neuronal activities has been tested in various experimental and simulated set-ups. For example, to explore the sufficiency of pairwise correlations or higher-order moments, or their predictive power for distribution tails [e.g. 34–48], and to characterize dynamical regimes [36, 49–51].

The probability distribution thus obtained, which includes the single-unit and pairwise statistics of the observation by construction, could help us to solve the background-correlation problem described above. In assigning to every observed activity pattern a probability, we obtain a measure of “surprise” for each such pattern; this surprise measure [e.g. 52, 53] is related to the logarithm of the probability and thus to Shannon’s entropy. Periods of activity with low probability correspond to large surprise: these patterns cannot be explained by the statistical properties that entered the construction of the probability distribution. In this way, we are able to effectively differentiate expected, less surprising events from those that are unexpected, surprising, and functionally meaningful.

Computing the maximum-entropy distribution from moment constraints—usually called the *inverse problem*–is simple in principle: it amounts to finding the maximum of a convex function. Hence optimization is straightforward [54, 55]. The maximum can be searched for with a variety of methods (downhill simplex, direction set, conjugate gradient, etc. [56, ch. 10]). The convex function, however, involves a sum over exp(*N*) terms, where *N* is the number of neurons. For 60 neurons, that is roughly twice the universe’s age in seconds, and modern technologies enable us to record *hundreds* of neurons simultaneously [57–60]. Owing to the combinatorial explosion for such large numbers of neurons, the convex function cannot be calculated, not even numerically. It is therefore “sampled”, usually via Markov-chain Monte Carlo techniques [61, 62]. In neuroscience the *Glauber dynamics*, also known as Gibbs sampling [61, 63, chap. 29], is usually chosen as the Markov chain whose stationary probability distribution is the maximum-entropy one. *Boltzmann learning* [64] is the iterative combination of sampling and search for the maximum, and is still considered the most precise method of computing a maximum-entropy distribution. Alternatively one may try to approximate the convex function by an analytic expression, as done with the mean-field [65, 66], Thouless-Anderson-Palmer [66, 67], and Sessak-Monasson [68, 69] approximations. The goodness of these approximations is usually checked against a Boltzmann-learning calculation [cf. 45].

Moment-constrained maximum-entropy models have also been used [70, 71] as generators of surrogate data, again via a Glauber dynamics. Such surrogates are used to implement a null hypothesis to estimate the statistical significance level of correlations between spike trains [70, 72–77].

The pairwise maximum-entropy model is applicable to *experimentally* recorded activities of populations of a couple hundreds neurons at most, so far; but its success, or lack thereof, cannot be automatically extrapolated to larger population sizes. Roudi et al. [78] gave evidence that the maximized Shannon entropy and other comparative entropies of such a model may present qualitatively different features above a particular population size. In the present paper we discuss a feature of the pairwise maximum-entropy model that may be problematic or undesirable: the marginal distribution for the population-averaged activity becomes *bimodal*, and one of the modes may peak at high activities. In other words, maximum-entropy claims that the population should fluctuate between a regime with a small fraction of simultaneously active neurons, and another regime with a higher fraction of simultaneously active neurons; the fraction of the second regime can be as high as 90%. This feature of the maximum-entropy model has been observed before in several theoretical studies that assumed a homogeneous neuronal population [see e.g. 34, 41, 79, 80].

Our analysis has several points in common with Bohte & al.’s [34]. Bohte et al. wanted to see whether a maximum-entropy distribution can correctly predict the distribution of total activity, given only firing rates and pairwise correlations from a simulated network model as constraints. They found that both the simulation and the maximum-entropy model yield a bimodal distribution of total activity within particular ranges of firing rates and correlations. The fundamental difference from our work is that our experimental data do not show a bimodal distribution, but the maximum entropy model wrongly predicts such bimodality from the measured rates and correlations. More quantitatively, the pairwise correlation found in our data is much lower than that reported in Bohte et al.; in particular, it seems to belong to the range in which their simulation yielded a unimodal distribution [34, p. 169]. Their simulations therefore seems to corroborate that a second mode is biologically implausible in our correlation regime.

Amari & al. [79] notice the appearance of bimodal distributions for the averaged activity and analyse some of their features in the *N* → ∞ limit. Their focus is on the correlations needed to obtain a “widespread” distribution in that limit. Our focus is on the bimodality appearing for large but finite *N*, and we find some mathematical results that might be at variance with Amari & al.’s. They seem to find [79, p. 135] that the Dirac-delta modes are at values 0 and 1; we find that they can appear also strictly within this range. They say [79, p. 138] that the “bigger peak” dominates as *N* → ∞; we find that the height ratio between the peaks is finite and depends on the single and pairwise average activity, and for our data is about 2000 as *N* → ∞—an observable value for recording lengths achievable in present-day experiments.

We provide evidence that the bimodality of the pairwise model is bound to appear in applications to populations of more than a hundred neurons. It renders the pairwise maximum-entropy model problematic for several reasons. First, in neurobiological data the coexistence of two regimes appears unrealistic—especially if the second regime corresponds to 90% of all units being simultaneously active within few milliseconds. Second, two complementary problems appear with the Glauber dynamics and the Boltzmann-learning used to find the model’s parameters. In the Glauber dynamics the activity alternately hovers about either regime for *sustained* periods, which is again unrealistic and rules out this method to generate meaningful surrogate data. In addition, the Glauber dynamics becomes practically *non-ergodic*, and the pairwise model *cannot be calculated at all* via Boltzmann learning or via the approximations previously mentioned [cf. 62, S 2.1.3; 61, chap. 29]. This case is particularly subtle because it can go undetected: *the non-ergodic Boltzmann learning yields a distribution that is not the maximum-entropy distribution one was looking for*.

Bohte & al. [34] remark that their neuronal-network simulation had to incorporate one inhibitory neuron, with the effect of “curtailing population bursts” [34, p. 175], because “the absence of inhibitory neurons makes a network very quickly prone to saturation” [34, p. 162]. This is something that a standard maximum-entropy distribution cannot do, hence a limitation in its predictive power. It is intuitively clear that lack of inhibition and bimodality are related problems: we show this in section “*Intuitive understanding of the bimodality: Mean-field picture*” using a simple mean-field analysis.

In the present work we propose a modified maximum-entropy model; more precisely, we propose a *reference probability measure* to be used with the method of *maximum relative entropy* [e.g. 31, 81] (also called minimum discrimination information [82]; see [83] for a comparison of the two entropies). The principle and reference measure can be used with pairwise or higher-order constraints; standard maximum-entropy corresponds to a uniform measure. The proposed reference measure, presented in section “*Inhibited maximum-entropy model*”, solves three problems at once: (1) it leads to distributions without unrealistic modes and eliminates the bistability in the Glauber dynamics; (2) it leads to a maximum-entropy model that can be calculated via Boltzmann learning; (3) it can also “rescue” interesting distributions that otherwise would have to be discarded because incorrect. The reference measure we propose is neurobiologically motivated. It is a minimal representation of the statistical effects of inhibition naturally appearing in brain activity, and directly translates Bohte & al’s device of including one inhibitory neuron in the simulated network. Moreover, the reference measure has a simple analytic expression and the resulting maximum-entropy model *is still the stationary distribution of a particular Glauber dynamics*, so that it can also be used to generate surrogate data.

In the final “*Discussion*” we argue that the use of such a measure is not just an ad hoc solution, but a choice required by the underlying biology of neuronal networks: the necessity of non-uniform reference measures is similarly well-known in other statistical scientific fields, like radioastronomy and quantum mechanics.

The plan of this paper is the following: after some mathematical and methodological preliminaries, we show the appearance of the bimodality problem in the maximum-entropy model applied to an experimental dataset of the activity of 159 neurons recorded from macaque motor cortex. Then we use an analytically tractable homogeneous pairwise maximum-entropy model to give evidence that the bimodality problem will affect larger and larger ranges of datasets as the population size increases. We show that typical experimental datasets of neural activity are prone to this problem.

We then investigate the underlying biological causes of the bimodality problem and propose a way to eliminate it: using a minimal amount of inhibition in the network, represented in a modified Glauber dynamics that includes a minimal *asymmetric inhibition*. We show that this correction corresponds to using the method of maximum entropy with a different reference measure, as discussed above, and that the resulting maximum entropy distribution is the stationary distribution of a modified Glauber dynamics. We finally bring to a close with a summary, a justification and discussion of the maximum-entropy model with the modified reference measure, and a comparison with other statistical models used in the literature.

## Results

### Preliminaries: Maximum-entropy models and Glauber dynamics

Our study uses three main mathematical objects: the pairwise maximum-entropy distribution, a “reduced” pairwise maximum-entropy distribution, and the Glauber dynamics associated with them. We review them here; some remarks about their range of applicability are given in. Towards the end of the paper we will introduce an additional maximum-entropy distribution.

#### Pairwise maximum-entropy model

Neuronal activity is modelled as a set of sequences of spikes of *N* neurons during a finite time interval [0, *T*]. These spike sequences are discretized: we divide the time interval into *n* bins of identical length Δ equal to *T*/*n*, indexed by *t* in 1, …, *n*. For each neuron *i*, the existence of one or more spikes in bin *t* is represented by *s*_*i*_(*t*) = 1, and lack of spikes by *s*_*i*_(*t*) = 0. With this binary representation, the activity of our population at time bin *t* is described by a vector: ***s***(*t*) ≔ (*s*_*i*_(*t*)). We will switch freely between vector and component notation for this and other quantities.

*Time* averages are denoted by a circumflex: $\hat{\cdot }$. The time-averaged activity of neuron *i* is denoted by *m*_*i*_:

$$\begin{array}{c} m_{i}≔\hat{s_{i}\left(t\right)}≔\frac{1}{T}\;\sum_{t=1}^{n}\;s_{i}\left(t\right), \end{array}$$ (1)

and the time average of the product of the activities of the neuron pair *ij*, called raw correlation or *coupled activity*, is denoted by *g*_*ij*_:

$$\begin{array}{c} g_{ij}≔\hat{s_{i}\left(t\right)\;s_{j}\left(t\right)}≔\frac{1}{T}\;\sum_{t=1}^{n}\;s_{i}\left(t\right)\;s_{j}\left(t\right). \end{array}$$ (2)

These time averages will be used as constraints for the maximum-entropy model.

The *pairwise* maximum-entropy statistical model [34, 35, 39, 40] assigns a time-independent probability distribution for the population activity ***s***(*t*) of the form (time is therefore omitted in the notation):

$$\begin{array}{c} \begin{array}{ccc} P_{\text{p}}\left(s|h,J\right) & = & \frac{1}{Z_{\text{p}}\left(h,J\right)}\exp(\sum_{i}\;h_{i}s_{i}+\sum_{i<j}\;J_{ij}s_{i}s_{j}),\\ Z_{\text{p}}\left(h,J\right) & ≔ & \sum_{s}\;\exp(\sum_{i}\;h_{i}s_{i}+\sum_{i<j}\;J_{ij}s_{i}s_{j}); \end{array} \end{array}$$ (3)

the Lagrange multipliers ***h***(***m***, ***g***) and ***J***(***m***, ***g***) are determined by enforcing the equality of the time averages Eqs (1) and (2) with the single- and coupled-activity expectations, with their definitions

$$\begin{array}{c} E_{\text{p}}(s_{i})≔\sum_{s}\;s_{i}\;P_{\text{p}}\left(s\right),\;E_{\text{p}}(s_{i}s_{j})≔\sum_{s}\;s_{i}s_{j}\;P_{\text{p}}\left(s\right) \end{array}$$ (4)

that is, enforcing

$$\begin{array}{c} E_{\text{p}}(s_{i})=m_{i}\;\text{and}\;E_{\text{p}}(s_{i}s_{j})=g_{ij}. \end{array}$$ (5)

By introducing the covariances ***c*** and Pearson correlation coefficients ***ρ***,

$$\begin{array}{c} \begin{array}{ccc} c_{ij} & ≔ & E(s_{i}s_{j})-E(s_{i})E(s_{j}),\\ \rho_{ij} & ≔ & \frac{c_{ij}}{\sqrt{\left[E_{\text{p}}(s_{i}^{2})-E_{\text{p}}{\left(s_{i}\right)}^{2}\right]\;\left[E_{\text{p}}(s_{j}^{2}-E_{\text{p}}{\left(s_{j}\right)}^{2}\right]}}, \end{array} \end{array}$$ (6)

the constraints above are jointly equivalent to

$$\begin{array}{c} E_{\text{p}}(s_{i})=m_{i}\;\text{and}\;c_{ij}=g_{ij}-m_{i}m_{j} \end{array}$$ (7)

or

$$\begin{array}{c} E_{\text{p}}(s_{i})=m_{i}\;\text{and}\;\rho_{ij}=\frac{g_{ij}-m_{i}m_{j}}{\sqrt{\left(m_{i}-m_{i}^{2}\right)\;\left(m_{j}-m_{j}^{2}\right)}}. \end{array}$$ (8)

The maximum-entropy distribution is unique if the constraints are convex. The covariance constraints *c*_*ij*_ = *g*_*ij*_ − *m*_*i*_*m*_*j*_ alone are not convex. In this case, uniqueness has to be checked separately [54, 55, 84]. On the other hand, the constraints E_p_(*s*_*i*_) = *m*_*i*_ and E_p_(*s*_*i*_*s*_*j*_) = *g*_*ij*_ are separately convex, thus their conjunction [*E_p_*(*s*_*i*_) = *m*_*i*_] ∧ [*E*_*p*_(*s*_*i*_*s*_*j*_) = *g*_*ij*_] is convex too. The bijective correspondence of the latter with [*E*_*p*_(*s*_*i*_) = *m*_*i*_] ∧ [*c*_*ij*_ = *g*_*ij*_ − *m*_*i*_*m*_*j*_] guarantees that the latter set of constraints is convex as well. What we have said about the covariances ***c*** also holds for the correlations ***ρ***.

#### Reduced maximum-entropy model

If the time-averaged activities ***m*** are homogeneous, i.e. equal to one another and to their population average $\bar{m}$, and the *N* (*N* − 1)/2 time-averaged coupled activities ***g*** are also homogeneous with population average $\bar{g}$, $\bar{g}≔\frac{2}{N\;(N-1)}\sum_{i<j}g_{ij}$, then the pairwise maximum-entropy distribution has homogeneous Lagrange multipliers by symmetry: *h*_*i*_ = *h*_r_ and *J*_*ij*_ = *J*_r_. It reduces to the simpler and analytically tractable form

$$\begin{array}{c} \begin{array}{ccc} P_{\text{r}}\left(s|h_{\text{r}},J_{\text{r}}\right) & = & \frac{1}{Z_{\text{r}}\left(h_{\text{r}},J_{\text{r}}\right)}\exp\left[h_{\text{r}}N\bar{s}+\frac{1}{2}J_{\text{r}}\;N\bar{s}\;\left(N\bar{s}-1\right)\right],\\ Z_{\text{r}}\left(h_{\text{r}},J_{\text{r}}\right) & ≔ & \sum_{s}\exp\left[h_{\text{r}}N\bar{s}+\frac{1}{2}J_{\text{r}}\;N\bar{s}\;\left(N\bar{s}-1\right)\right], \end{array} \end{array}$$ (9)

which assigns equal probabilities to all those activities ***s*** that have the same *population-averaged activity* $\bar{s}$, defined as

$$\begin{array}{c} \bar{s}≔\frac{1}{N}\;\sum_{i=1}^{N}\;s_{i},\\ N\bar{s}\in \{0,1,2,\ldots ,N\}. \end{array}$$

We denote *population* averages that are normalized to the number of neurons by an overbar: $\bar{\cdot }$. Then the quantity $N\bar{s}$ represents the sum of the activities of all neurons in the population at time bin *t*; we call it *total activity*, and its plot is called “population time-histogram” in some works [cf 85, 86].

In this homogeneous case, the values of the multipliers appearing in Eq (9) are equal to their population averages: $h_{i}=h_{\text{r}}=\frac{1}{N}\;\sum_{i}\;h_{i}$ and $J_{ij}=J_{\text{r}}=\frac{2}{N\;(N-1)}\sum_{i<j}J_{ij}$. This distribution hence contains only information about how many neurons $N\bar{s}$ are active at any given point in time, but not the particular composition ***s*** of active neurons. This simpler distribution can therefore also be interpreted as an approximation of the pairwise maximum-entropy one, achieved by disregarding population inhomogeneities of the constraints *m*_*i*_ and *g*_*ij*_. But it is also an exact maximum-entropy distribution in its own right, obtained by only constraining the expectations for the population averages of the single and coupled activities,

$$\begin{array}{c} \sum_{i}\;s_{i}=N\bar{s},\;\sum_{i<j}\;s_{i}s_{j}=N\bar{s}\;\left(N\bar{s}-1\right)/2, \end{array}$$

to be equal to their measured time averages

$$\begin{array}{c} E_{\text{r}}(N\bar{s})=N\bar{m}\;\text{and}\;E_{\text{r}}(N\bar{s}\;\left(N\bar{s}-1)\right)=\frac{N\;(N-1)}{2}\bar{g}≔\sum_{i<j}\;g_{ij}. \end{array}$$ (10)

For this reason we call the model Eq (9) a *reduced* pairwise maximum-entropy model. But in the inhomogeneous case the multipliers of the reduced model are *not* equal to the averages of the pairwise one: $h_{\text{r}}\neq \frac{1}{N}\;\sum_{i}\;h_{i}$, $J_{\text{r}}\neq \frac{2}{N\;(N-1)}\;\sum_{i<j}\;J_{ij}$.

It is straightforward to derive the probability distribution for the population average $\bar{s}$ in this model, owing to its symmetry: the total number of active neurons in the population is $N\bar{s}$, and there are (NNs¯) equally probable ways in which this is possible, each with probability by Eq (9). Therefore,

Pr(s¯|hr,Jr)=1Zr(hr,Jr)(NNs¯)exp[hrNs¯+12JrNs¯(Ns¯-1)],Zr(hr,Jr)≔∑s¯(NNs¯)exp[hrNs¯+12JrNs¯(Ns¯-1)]. (11)

This probability distribution $P_{\text{r}}\left(\bar{s}\right)$ can, in turn, also be obtained applying a maximum-relative-entropy principle [31, 83], i.e. minimizing the relative entropy (or discrimination information)

$$\begin{array}{c} H\left(P,P_{0}\right)≔\sum_{\bar{s}}\;P\left(\bar{s}\right)\ln\frac{P(\bar{s})}{P_{0}\left(\bar{s}\right)} \end{array}$$ (12)

of $P(\bar{s})$ with respect to the reference distribution P0(s¯)=2-N(NNs¯) while constraining its first two moments, or equivalently its first two factorial moments [87] $\left(E(N\bar{s}),E(N\bar{s}\;\left(N\bar{s}-1\right)/2)\right)$.

It is easy to see that in this model, by symmetry, we also have

$$E_{\text{r}}(s_{i})=E_{\text{r}}(\bar{s}),\;E_{\text{r}}(s_{i}s_{j})=E_{\text{r}}\;(\frac{N\bar{s}\;\left(N\bar{s}-1\right)}{N\;(N-1)}),$$ (13)

$$c_{ij}=\bar{c}={\text{E}}_{\text{r}}\left(\frac{N\bar{s}\left(N\bar{s}-1\right)}{N\left(N-1\right)}\right)-{\text{E}}_{\text{r}}{\left(\bar{s}\right)}^{2},\;\rho_{ij}=\bar{\rho }=\frac{\bar{c}}{{\text{E}}_{\text{r}}\left(\bar{s}\right)-{\text{E}}_{\text{r}}{\left(\bar{s}\right)}^{2}},$$ (14)

and $\left(E_{\text{r}}(\bar{s}),E_{\text{r}}(\frac{N\bar{s}\;\left(N\bar{s}-1\right)}{N\;(N-1)})\right)$, $\left(E_{\text{r}}(\bar{s}),\bar{c}\right)$, $\left(E_{\text{r}}(\bar{s}),\bar{\rho }\right)$ are equivalent sets of constraints ($\bar{c}$ and $\bar{\rho }$ by themselves are not convex).

This reduced maximum-entropy model is mathematically very convenient because the Lagrange multipliers *h*_r_, *J*_r_ can be easily found numerically (with standard convex-optimization methods like downhill simplex, direction set, conjugate gradient, etc. [56, ch. 10]) with high precision even for large (e.g. thousands) population sizes *N*.

The pairwise and reduced models are very similar to the *Gaussian ensemble* of statistical mechanics [88–90, and refs therein], in which the mean and variance of a system’s energy are constrained; it is intermediate in properties between the canonical and microcanonical ensembles.

The maximum-entropy models reviewed above use *time-averaged* data. Their probabilities are therefore time-invariant; they are stationary statistical models.

#### Glauber dynamics and Boltzmann learning

The normalization *Z*_p_(***h***, ***J***) appearing in the probability distribution Eq (3) requires the summation over 2^*N*^ states, typically in the range of *N* ≈ 100. This calculation may require prohibitive amounts of time; we need a way to calculate the distribution that avoids the computation of the normalization. The probability *P*_p_(***s***|***h***, ***J***) Eq (3) is identical in form to the stationary distribution of a Markov chain **s**(*t*) ↦ **s**(*t* + 1), the so-called asynchronous Glauber dynamics [63]. If this dynamics is ergodic, after an initial transient it generates states with a relative frequency distribution approximately equal to the stationary one. In this context the symmetric Lagrange multipliers ***J*** are sometimes also referred to as “couplings”, in analogy to synaptic interactions between the units. The Lagrange multipliers ***h*** are referred to as “biases” and may be interpreted as either a threshold or external input controlling the base activity of individual neurons. The temporal sequence of states produced by the Glauber dynamics is predominantly controlled by the time constant of the update rule and in general does not reflect the temporal evolution of the neuronal population activity.

If we assume that our uncertainty about the *evolution* of the population activity can be modelled by the Glauber dynamics of a binary network, we can employ the Glauber dynamics, with ***h***, ***J*** parameters determined by the constraints Eq (5), to generate surrogate data that would allow us to implement a null-hypothesis for a statistical test, that includes the average activities and pairwise correlations as observed in the data. This procedure requires that the dynamics should be ergodic, sampling the entire state space. Otherwise, time averages obtained from the surrogate would not coincide with those of our experimentally observed data.

The Glauber dynamics is used as the sampling step in Boltzmann learning [64], as mentioned in the “*Introduction*”, to find the parameters ***h***(***m***, ***g***) and ***J***(***m***, ***g***) of the maximum entropy distribution Eq (3) having mean activities ***m*** and raw pairwise correlations *g*. Starting with some values of the multipliers $\hat{h}$ and $\hat{J}$, the distribution is sampled by the Glauber dynamics and the its averages of the single activity $\hat{m}$ and the coupled activity $\hat{g}$ are found for the current values of the multipliers. The latter are then adjusted in relation to the mismatch $\left|\hat{m}-m\right|$, $\left|\hat{g}-g\right|$ between the sampled averages and the required values from the experimental data. A new sampling is then performed, and so on until the mismatch lies below a prescribed accuracy.

### The problem: Bimodality, bistability, non-ergodicity

We first show how the bimodality problem subtly appears with a set of experimental data, then explore its significance for larger population sizes and other samples of experimental data of brain activity.

#### Experimental data: Preliminary approximations

The data, provided by A. Riehle and T. Brochier (INT, CNRS-AMU Marseille, France), consist of the activity of a population of *N* = 159 single neurons recorded from motor cortex of macaque monkey for 15 minutes, using a 100-electrode “Utah” array as described in [60], but with a different behavioural design: here the monkey was awake and alert, but did not perform any task during the recording. This behavioural protocol is chosen for retrieving “resting” (or “ongoing”) state [91] data to characterize the “ground” state, in contrast to a task or functional state.

**Fig 1:**
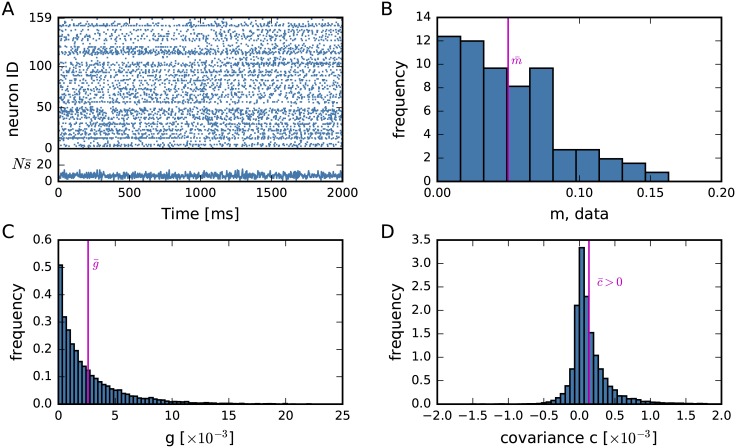
Experimental data and their empirical first- and second-order statistics. (**A**) Example raster display (snippet of 2s from the total data of 15 min) of *N* = 159 parallel spike recordings of macaque monkey during a state of “ongoing activity”. The experimental data are recorded with a 100-electrode “Utah” array (Blackrock Microsystems, Salt Lake City, UT, USA) with 400 *μ*m interelectrode distance, covering an area of 4 × 4 mm^2^ (session: s131214-002). The total activity $N\bar{s}\left(t\right)$ shows the number of active neurons within each time bin *t* of width Δ = 3 ms. (**B**) Population distribution of the time-averaged activities *m*_*i*_ (in spikes/Δ) of each of the neurons *i*, Eq (1). The vertical line marks the population average $\bar{m}=0.0499$. (**C**) Population distribution of the time-averaged raw correlations (coupled activities) *g*_*ij*_, Eq (2). The vertical line marks the population average, $\bar{g}≔\frac{2}{N\;(N-1)}\;\sum_{i<j}g_{ij}=0.00261$. (**D**) Population distribution of the covariances *c*_*ij*_ = *g*_*ij*_ − *m*_*i*_*m*_*j*_. The vertical line marks the slightly positive population average, $\bar{c}≔\frac{2}{N\;(N-1)}\;\sum_{i<j}c_{ij}=0.000135$. Histograms bins in B, C, D are computed with Knuth’s rule [92] and calculated over the full 15-minute long recording. Data courtesy by A. Riehle and T. Brochier.

Fig 1A shows a raster plot (2s out of 15min for better visibility) of the activities ***s***(*t*) of all recorded neurons. The population-averaged activity $\bar{s}\left(t\right)$ for this period is shown underneath. The distributions of the time-averaged single and coupled activities *m*_*i*_, *g*_*ij*_, and the corresponding empirical covariances *c*_*ij*_ measured in the full data set of 15 min are shown in panels B, C, D. The population averages of these time-averaged quantities are

$$\begin{array}{c} \bar{m}=0.0499,\;\bar{g}=0.00261,\;\bar{c}=0.000135,\;\bar{\rho }=0.00319. \end{array}$$ (15)

As discussed in the previous section, the pairwise maximum-entropy model is a stationary statistical model. If we intended to analyse the dataset above with this model for a specific purpose—for example, characterizing a “ground state” of behavioural activity—then we would have to assess whether a stationary model would really suit these particular data and purpose. It would not be suitable, for example, to model transient aspects of neural activity. Our goal, however, is rather to analyse the general presence of bimodality in the model for data with ranges and orders of magnitude typical of recorded brain activity. In section “*Bimodality of pairwise models for massively parallel data*” we will see that our conclusions regarding bimodality are valid even if the population average $\bar{m}$ is doubled or halved and if the Pearson correlation $\bar{\rho }$ becomes ten times smaller or larger; thus amply valid within any non-stationarity corrections [93]. Moreover, stationary maximum-entropy models have also been used with highly fluctuating data, e.g. from retinal cells [e.g. 35, 40, 43, 94], with the purpose of analysing some of their information-theoretic properties rather than of modelling the data. For these reasons, and also for brevity, we do not address stationarity analyses and corrections in the present work.

We now need to find the parameters ***h***(***m***, ***g***) and ***J***(***m***, ***g***) of the maximum entropy distribution Eq (3) so that the mean activities ***m*** and the raw pairwise correlations ***g*** correspond to those measured in the data shown in Fig 1B and 1C. We try to find these parameters via Boltzmann learning with a Glauber dynamics, as explained in “*Glauber dynamics and Boltzmann learning*”.

**Fig 2:**
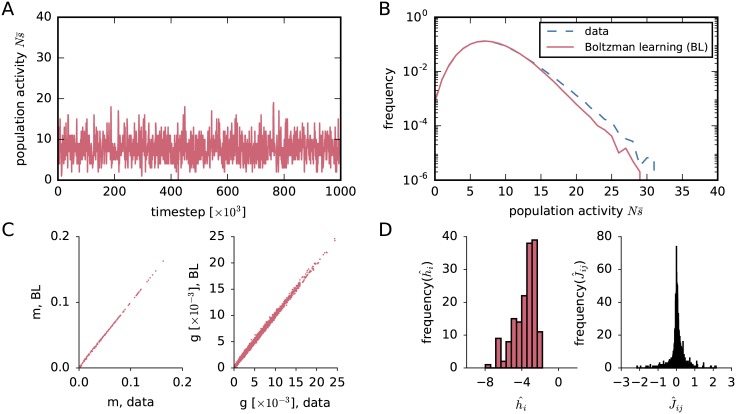
Preliminary results from Boltzmann learning. (**A**) Evolution of the total-population activity $N\bar{s}\left(t\right)$ of *N* = 159 neurons produced by the Glauber dynamics (implemented in NEST [96]; see section “*Simulation of Glauber dynamics with NEST*”) with 10^6^ steps, during the sampling phase of the last Boltzmann-learning iteration. (**B**) Red, solid: Preliminary approximation of the probability distribution of the population-averaged activity, obtained via Boltzmann learning. Blue, dashed: empirical distribution of the population-averaged activity from the dataset shown in A. (**C**) Preliminary values of the time averages *m*_*i*_ and *g*_*ij*_ obtained from Boltzmann learning described in (A), versus the experimental ones shown in Fig 1. (**D**) Preliminary approximations of the population distributions of the Lagrange multipliers ${\hat{h}}_{i}$ and ${\hat{J}}_{ij}$ (associated with the averages *m*_*i*_ and *g*_*ij*_) obtained via Boltzmann learning. Histogram bins in D are computed with Knuth’s rule [92].

We choose the sampling phase of the Boltzmann learning to have 10^6^ timesteps; an example is shown in Fig 2A. This number of timesteps is large compared to Roudi et al. [45] (200 timesteps) or Broderick et al. [95] (400 timesteps). The preliminary approximations $\left({\hat{h}}_{i},{\hat{J}}_{ij}\right)$ of the Lagrange multipliers obtained in this way are shown in Fig 2D. The final single and coupled activities are shown in Fig 2C, compared to the experimental data. The first and second moments are highly correlated with the experimental ones and seem to describe the data well. The preliminary approximation of the population-average probability distribution is shown in red in Fig 2B. Its tail disagrees with that of the empirical frequency distribution (dashed); but, before discussing about curve-fitting properties, we want to make sure that our initial approximations are correct. In fact, we shall now see that these preliminary approximations are *not* correct in this case.

#### Appearance of bimodality

**Fig 3:**
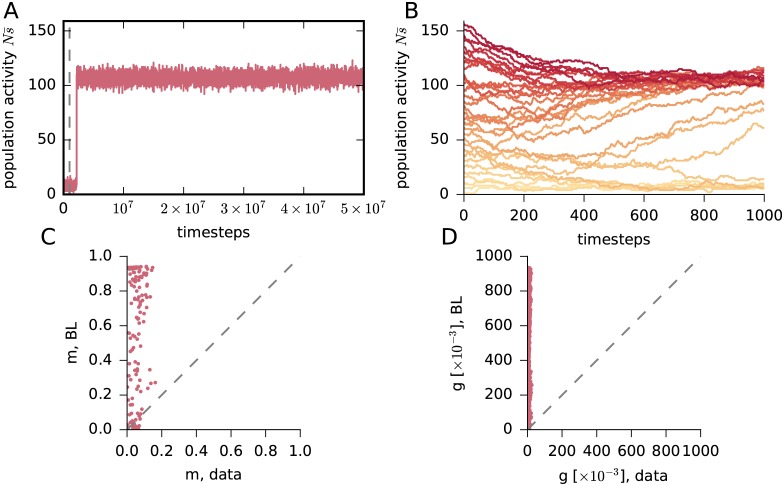
Longer sampling: Bistability. (**A**) Evolution of the total activity $N\bar{s}\left(t\right)$ produced by Glauber dynamics, as in Fig 2A, but with 5 × 10^7^ steps. The dashed grey line marks the end of the previous shorter sampling of Fig 2A. (**B**) Evolutions of the total-population activity $N\bar{s}\left(t\right)$ obtained from several instances of Glauber dynamics. Each instance starts with a different value of the initial total activity $N\bar{s}\left(0\right)$, i.e. with $N\bar{s}\left(0\right)$ initially active neurons (chosen at random), and is represented by a different red shade, from $N\bar{s}\left(0\right)=0$ (light red) to $N\bar{s}\left(0\right)=N$ (dark red). Note the two convergence basins, one at $N\bar{s}\approx 100$ and one at $N\bar{s}\approx 15$. (**C**, **D**) New values of the time averages *m*_*i*_ and *g*_*ij*_ versus the experimental ones. These new values are obtained from the longer Glauber dynamics described in (A) using the values of the Lagrange multipliers $\hat{h_{i}},\;\hat{J_{ij}}$ shown in Fig 2D, obtained from the previous Boltzmann learning. The plots clearly show that the values of $\hat{h_{i}},\;\hat{J_{ij}}$ found with the Boltzmann learning are not the ones yielding the constraints *m*_*i*_, *g*_*ij*_.

The preliminary results from Boltzmann learning do not show any inconsistency at this point. But now we sample the distribution for a much longer time: 5 × 10^7^ steps, to verify whether these approximations have truly converged. The result is shown in Fig 3A. We find that after roughly 2 × 10^6^ steps the whole population jumps to a high-activity regime and remains there until the end of the sampling. We have thus discovered that:

- our preliminary approximations of the Lagrange multipliers are wrong; their mismatch is shown in Fig 3C–3D;
- our preliminary approximation of the pairwise distribution, Fig 2B, is therefore wrong in two ways: it does not correspond to the (wrong) approximations of the Lagrange multipliers, and is not a pairwise maximum-entropy distribution;
- the Glauber dynamics has an additional metastable high-activity regime.

It is legitimate to wonder whether there are other metastable regimes. To test this possibility we start the dynamics with different numbers of initially active neurons. Two metastable regimes are observed (see Fig 3B): one at high activity and one at low activity. This means that the distribution associated with the initial, wrong approximations of the Lagrange multipliers of Fig 2D is *bimodal*, not unimodal as Fig 2B seemed to show.

Note that choosing as initial condition for the sampling procedure a state in the low-activity regime will not prevent the Glauber dynamics from entering the high activity state. As shown in Fig 3B, the system may still spontaneously transition into the high activity regime with a small but not negligible probability.

What do these facts imply? Let us recall that our primary goal is to model the data with an inhomogeneous pairwise maximum-entropy distribution. Boltzmann learning is just a procedure to find this distribution. This procedure explores the space of distributions in a particular way to find the correct one. What we just found says that this procedure entered a region of bimodal distributions in such space and got stuck there, without finding the correct distribution yet.

We must reflect on three main issues:

1. Boltzmann learning becomes impractically slow when it enters the bimodal region, because the Glauber dynamics that is part of this procedure becomes almost *non-ergodic*. Therefore it is an inefficient method to find the pairwise maximum-entropy distribution. Non-ergodicity is a known phenomenon in Monte Carlo methods; its solution requires longer sampling times or algorithms different from Glauber sampling [61, 62, 97, 98]. This problem also appears, for example, in the calculation of extensive parameters in finite-size statistical mechanics in phase-transition regimes [99]. Note that bistability is not an *effect* of longer sampling; rather, longer sampling becomes a *necessity* because of bistability. This bistability is an inherent mathematical phenomenon caused by the positivity of the average correlations together with the large number *N* of units, as shown in sections “*Bimodality ranges and population size*” and “*Bimodality of the inhomogeneous model for large N*”.
  We could then try to use alternative procedures to find our desired distribution. The Thouless-Anderson-Palmer approximation [66, 67] and the Sessak-Monasson approximation [68, 69], for example, have been successfully used in the literature for this purpose. But unfortunately we find that these two approximations do not give the correct distribution either: properly sampled, the distributions they yield do not match the correlations and means of our data, just as in Fig 3C–3D. Evidently our data lie outside the domains of validity of these two approximations. Notably, the incorrect distributions given by these two approximations are also bimodal.
2. There may be one more problem ahead, though. Never mind that the procedures we know do not work; suppose we find a procedure that gives us the inhomogeneous pairwise distribution we are seeking. What should we do if this distribution turns out to be bimodal with a second mode at unrealistically high activities? Its Glauber dynamics would be bistable, yielding *sustained* periods of high activity, which would not be useful to generate meaningful, realistic surrogate data. Would we still be willing to use this maximum-entropy statistical model, or should we reject it altogether? And is the appearance of a bimodal distribution only peculiar to our data, or a more widespread feature of brain-activity data?
3. Besides, it is a pity that our initial approximation of the maximum-entropy distribution, Fig 2B, was incorrect. We had found *some* probability distribution, but it was *not* a pairwise maximum-entropy distribution; yet that distribution was modelling our data in an interesting way—and such modelling is our priority. This situation can be confusing, so let us explain it with an analogy. Imagine that we have some datapoints and we say “we want to fit the points with a *parabola*”. An incorrect fitting algorithm, however, gives us a curve that is not parabola. If this curve covers the datapoints in an interesting way, we may want to investigate what kind of curve it is. It could turn out to be a *hyperbole*, for example. We may then want to broaden our point of view and say “we want to fit the points with a *quadric*”, and use that curve. (We should still fix the fitting algorithm, though.) In our case, can we find out more about the probability distribution of Fig 2B? Could it also be a member of an extended maximum-entropy family for example?

We will shortly show that there is one solution that addresses these three issues all at once. We think, in fact, that it also addresses a fourth issue of maximum-entropy models, to be discussed later. We first analyse issues 2. and 3. in more detail; issue 1. above is subordinate to them.

#### Is the correct pairwise model bimodal?

We would like to know whether the correct maximum-entropy distribution, which we have not found yet, is also bimodal like its incorrect approximation.

We make an educated guess by examining the analytically tractable reduced maximum-entropy model *P*_r_, Eq (9). Using the population-averaged single and coupled activities as constraints, $E_{\text{r}}(\bar{s_{i}})=\bar{m}$ and $E_{\text{r}}(\bar{s_{i}s_{j}})=\bar{g}$ from Eq (15), we numerically find the Lagrange multipliers of the reduced model:

$$\begin{array}{c} h_{\text{r}}=-3.259,\;J_{\text{r}}=0.03859. \end{array}$$ (16)

Note that in this case there is no sampling involved: the distribution can be calculated analytically, and the values Eq (16) are correct within the numerical precision of the maximization procedure (interior-point method [56, chap. 10]). The values of the expected single and couple activities, re-obtained by explicit summation (not sampling) from the corresponding reduced maximum-entropy distribution, agree with the values Eq (15) to seven significant figures.

**Fig 4:**
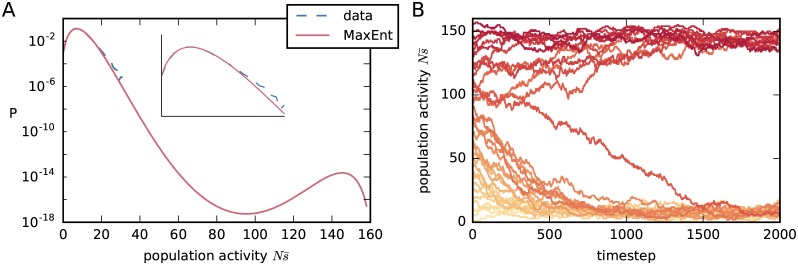
Reduced maximum-entropy model. (**A**) Red, solid: Probability distribution for the population-averaged activity, $P_{\text{r}}\left(\bar{s}\right)$ given by the reduced model for our dataset Eq (15); note the two probability maxima. Blue, dashed: empirical frequency distribution of the population-averaged activity from our dataset. (**B**) Population-averaged activities $\bar{s}\left(t\right)$ obtained from several instances of the Glauber dynamics associated with the reduced model, with homogeneous couplings, *J*_*ij*_ = *J*_r_, and biases, *h*_*i*_ = *h*_r_, of Eq (16). As in Fig 3, each instance starts with an initial population activity ***s***(0) having different values of population average $\bar{s}\left(0\right)$, and is represented by a different red shaded curve. The initial values range from $N\bar{s}\left(0\right)=0$ (light red) to $N\bar{s}\left(0\right)=N$ (dark red).

The resulting reduced maximum-entropy distribution for the population-averaged activity, $P_{\text{r}}\left(\bar{s}|h_{\text{r}},J_{\text{r}}\right)$, is shown in Fig 4A, together with the experimental frequency distribution of our data. Its corresponding Glauber dynamics with two metastable regimes is shown in Fig 4B. It shows a second maximum at roughly 90% activity.

A mathematically exact analysis of smaller subsets of our population with the inhomogeneous maximum-entropy model, and an analysis of the full population and large subsets of it with a reduced maximum-entropy model having higher-order constraints $E(N\bar{s})$, $E{\left(\left(N\bar{s}\right)\right)}^{2}$, $E(\frac{N\bar{s}\;\left(N\bar{s}-1\right)}{N\;(N-1)}-{\left(\frac{N\bar{s}\;\left(N\bar{s}-1\right)}{N\;(N-1)}\right)}^{2})$ (the latter corresponding to the variance of the second moments), show that if a reduced maximum-entropy model is bimodal, the full inhomogeneous model is also bimodal, with a heightened second mode shifted towards lower activities with respect to the reduced model.

The bimodality encountered in the Boltzman learning, the bimodality of the reduced maximum-entropy model, the bimodality of the full maximum-entropy model for small populations, and finally the bimodality for the reduced model with higher-order constraints, together constitute strong evidence that the correct pairwise maximum-entropy distribution for our data is bimodal. In section “*Bimodality of the inhomogeneous model for large N*” we prove that this must be true for large *N*, even if it were not true for our specific population size *N* = 159.

#### Bimodality ranges and population size

Next we want to address whether it is common or rare that the pairwise maximum-entropy method yields bimodal distributions for neuronal brain-activity data. For this purpose we first estimate the ranges of firing rates and correlations for which maximum-entropy yields a bimodal distribution; then we check whether typical experimental values fall within these ranges. We are particularly interested in how bimodality depends on the recorded population size *N*.

We again make an educated guess using the reduced maximum-entropy model, with distribution for the population average $\bar{s}$, $P_{\text{r}}\left(\bar{s}|h_{\text{r}},J_{\text{r}}\right)$, Eq (11). The distribution has two maxima if it has one minimum for some value ${\bar{s}}_{\text{m}}$, $0<{\bar{s}}_{\text{m}}<1$. An elementary study of the convexity properties (second derivative) of this distribution shows that it has one minimum at $0<{\bar{s}}_{\text{m}}<1$ if

$$\begin{array}{c} \frac{dP_{\text{r}}\left(\bar{s}|h_{\text{r}},J_{\text{r}}\right)}{d\bar{s}}{|}_{\bar{s}={\bar{s}}_{\text{m}}}=0,\;\frac{d^{2}P_{\text{r}}\left(\bar{s}|h_{\text{r}},J_{\text{r}}\right)}{d{\bar{s}}^{2}}{|}_{\bar{s}={\bar{s}}_{\text{m}}}>0,\;0<{\bar{s}}_{\text{m}}<1, \end{array}$$ (17)

**Fig 5:**
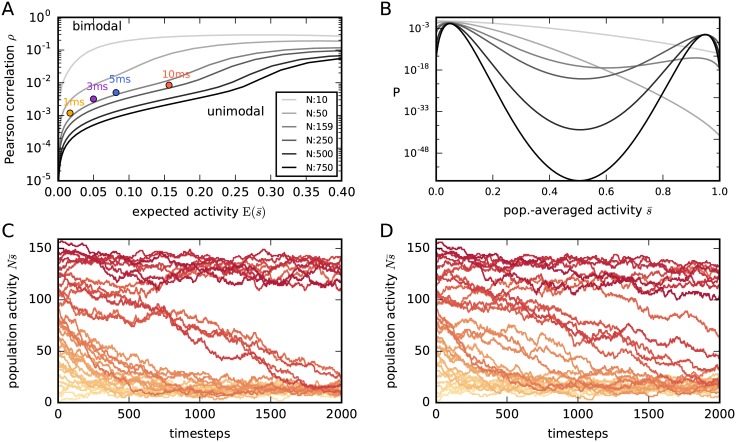
Bimodality ranges for the reduced model and effects of inhomogeneity. (**A**) The reduced maximum-entropy model Eq (9) yields a distribution $P_{\text{r}}\left(\bar{s}\right)$ that is either unimodal or bimodal, depending on the number of neurons *N* and the values of the experimental constraints $\left(E_{\text{r}}(\bar{s}),\bar{\rho }\right)$. Each curve in the plot corresponds to a particular *N* (see legend) and separates the values $\left(E_{\text{r}}(\bar{s}),\bar{\rho }\right)$ yielding a unimodal distribution, below the curve, from those yielding a bimodal one, above the curve. The curves are symmetric with respect to $E_{\text{r}}(\bar{s})=0.5$ (ranges $E_{\text{r}}(\bar{s})>0.4$ not shown). Note how the range of constraints yielding bimodality increases with *N*. Coloured dots show the experimental constraints from our dataset for different time-binnings with widths Δ = 1 ms, Δ = 3 ms, Δ = 5 ms, Δ = 10 ms: all these binnings yield a bimodal distribution. (**B**) Probability distributions of the reduced model for the population-averaged activity, $P_{\text{r}}\left(\bar{s}|N\right)$, obtained using the constraints Eq (15) from our data set (3 ms purple dot in panel A) and different *N* (same colour legend as panel A). (**C**) Population-averaged activities $\bar{s}\left(t\right)$ from several instances of Glauber dynamics, all with the same normally-distributed couplings *J*_*ij*_ and biases *h*_*i*_, with means as in Eq (16) and Fig 4B, and standard deviations *σ*(*J*_*ij*_) = 0.009, *σ*(*h*_*i*_) = 0.8. Each instance starts with an initial population activity ***s***(0) having different values of the population average $\bar{s}\left(0\right)$, and is represented by a different red shade, from $N\bar{s}\left(0\right)=0$ (light red) to $N\bar{s}\left(0\right)=N$ (dark red). Note how the basins of attraction of the two metastable regimes are wider than in the homogeneous case of Fig 4B. (**D**) The same as panel C, but with larger standard deviations *σ*(*J*_*ij*_) = 0.012, *σ*(*h*_*i*_) = 1.08; the jumps between the two metastable regimes become more frequent than in Fig 4B, indicating that the minimum between the modes becomes shallower as the inhomogeneity increases.

These conditions can be solved analytically and give the ranges of the multipliers (*h*_r_, *J*_r_) for which bimodality occurs, parametrically in (${\bar{s}}_{\text{m}}$, *J*_r_):

$$\{\begin{array}{c} 0<{\bar{s}}_{\text{m}}<1,\\ J_{\text{r}}>\Psi^{\prime }[1+(1-{\bar{s}}_{\text{m}})N]+\Psi^{\prime }(1+{\bar{s}}_{\text{m}}N),\\ h_{\text{r}}({\bar{s}}_{\text{m}},J_{\text{r}})=J_{\text{r}}/2-{\bar{s}}_{\text{m}}N\;J_{\text{r}}-\Psi [1+(1-{\bar{s}}_{\text{m}})N]+\Psi (1+{\bar{s}}_{\text{m}}N), \end{array}$$ (18)

where *Ψ*(*x*) ≔ *d* ln Γ(*x*)/*dx*, Γ being the Gamma function [100, chs 43–44]. We express the population-averaged activity $E_{\text{r}}(\bar{s})$ and the Pearson correlation $\bar{\rho }$, typically used in the literature, in terms of (*h*_r_, *J*_r_) using the definitions Eq (14) and the probability Eq (11). In this way we finally obtain the bimodality range for $\left(E_{\text{r}}(\bar{s}),\bar{\rho }\right)$, parametrically in (${\bar{s}}_{\text{m}}$, *J*_r_): the results are shown in Fig 5A for various values of the number of neurons *N*.

For each *N* we have a curve in the plane $E_{\text{r}}(\bar{s})$,$\bar{\rho }$. Values of $\left(E_{\text{r}}(\bar{s}),\bar{\rho }\right)$ above such curves yield a bimodal pairwise maximum-entropy distribution in the homogeneous case, for the corresponding population size *N*. The plot notably shows that the range of constraints yielding bimodality increases with *N*. Fig 5B displays the probability distribution of the population-averaged activity for the constraints from our dataset Eq (15) but different values of *N*. When *N* ≲ 150 the distribution has only one maximum at low activity, $\bar{s}\approx 0.0497$, and when *N* ≳ 150 a second probability maximum at high activity, $\bar{s}\approx 0.9502$, appears. The probability at this second maximum increases sharply until *N* ≈ 200 and thereafter maintains an approximately stable value, roughly 6000 times smaller than the low-activity maximum. The minimum between the two modes becomes deeper and deeper as we increase *N* above 200.

As mentioned in the previous section, exact studies with small samples and studies with large samples and a reduced model with higher-order constraints indicate that the high-activity maximum in the inhomogeneous case is even larger (roughly 2000 times smaller than the low-activity one when *N* = 1000) and shifted towards lower activities ($\bar{s}\approx 0.25$ when *N* = 1000).

This can also be seen by adding a Gaussian jitter to the multipliers of the reduced case *h*_*i*_ = *h*_r_, *J*_*ij*_ = *J*_r_, thereby making the model inhomogeneous. The results for small and large jitter are shown in Fig 5C–5D, respectively. The basin of attraction of the second metastable regime is shifted to lower activities, and transitions between the two metastable regimes become more likely for larger jitters. This means that inhomogeneity makes the minimum in between the two modes shallower.

The population-averaged activity and Pearson correlation of our data (violet “3 ms” point in Fig 5A) fall within the bimodality range.

#### Bimodality of pairwise models for massively parallel data

Having found the bimodality ranges of firing rates and correlations in section “*Bimodality ranges and population size*”, we now ascertain whether our dataset is a typical representative leading to bimodal pairwise distributions, or an outlier. We take as reference the data summarized in Table 1 of Cohen & Kohn [37], which reports firing rates and spike-count correlations r_SC_ for several experimental recordings of brain activity. The reported firing rates correspond to population-averaged activities $\bar{m}$ ranging between 0.02 and 0.25 if we use 3 ms time bins; thus our data are well within this range.

The values reported for the correlations *r*_SC_ in [37] are given for the spike counts measured in large time intervals: several hundred of milliseconds. We therefore need a coarse estimate of the Pearson correlation coefficient *ρ* that would be measured on a fine temporal scale of 3 ms in the same experiment. Both *r*_SC_ and *ρ* are particular cases of the Pearson correlation coefficient *r*_CCG_ of spike counts in a window *τ*, as introduced by Bair et al. [101, App. A]:

$$\begin{array}{c} \begin{array}{cc} r_{\text{CCG}\;ij}\left(\tau \right) & ≔\frac{E(\nu_{i}\left(\tau \right)\;\nu_{j}\left(\tau \right))-E(\nu_{i}\left(\tau \right))E(\nu_{j}\left(\tau \right))}{\sqrt{\left[E(\nu_{i}{\left(\tau \right)}^{2})-E({\nu_{i}\left(\tau \right))}^{2}\right]\;\left[E(\nu_{j}{\left(\tau \right)}^{2})-E({\nu_{j}\left(\tau \right))}^{2}\right]}},\\ & \text{where}\;\nu_{i}\left(\tau \right)≔\sum_{t=1}^{\tau }\;s_{i}\left(t\right), \end{array} \end{array}$$ (19)

i.e. *ν*_*i*_(*τ*) is the number of spikes of neuron *i* in the time window *τΔ*. Here *s*_*i*_(*t*) are the binary representations of the spike trains binned with bin width Δ, in line with section “*Pairwise maximum-entropy model*”. This metric also equals the area between times −*τΔ* and *τΔ* under the cross-correlogram of neurons *i* and *j* (stationarity is assumed), calculated on the fine temporal time scale Δ. The spike count correlation *r*_SC_ corresponds to *τ* = *n* ≡ *T*/*Δ*, and our Pearson correlation *ρ* to *τ* = 1. Several studies [we analysed: 101–105] report either measured values of *r*_CCG_(*τ*) for different windows *τ*, or measured cross-correlograms. We studied, one by one, all the measures reported in the cited studies and numerically found that each of them satisfies *ρ* ≳ *r*_SC_/20. We decide to take the lower bound $\bar{\rho }=\bar{r_{\text{SC}}}/20$ as a coarse estimate of *ρ* given *r*_SC_, because it leads to points as far away from bimodality as possible, i.e. because it is biased *against* our conjecture.

**Fig 6:**
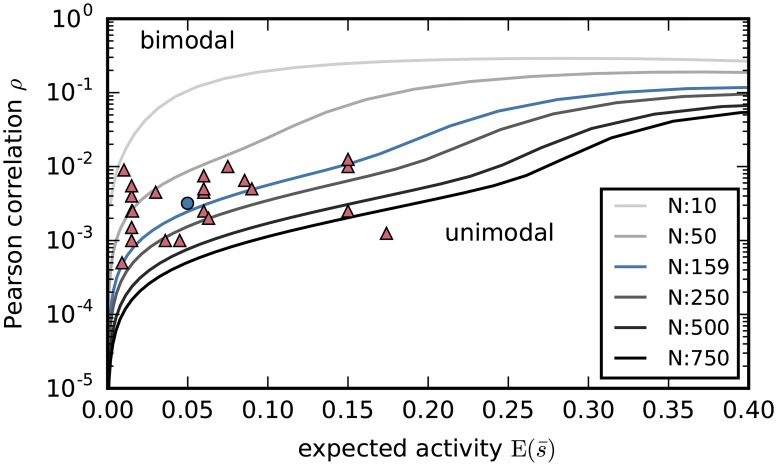
Bimodality for experimental data from neuroscientific literature. Mean activities and correlations $\left(E_{\text{r}}(\bar{s}),\bar{\rho }\right)$ inferred from experimental data reported in Cohen & Kohn [37, Table 1], plotted upon the curves separating bimodal from unimodal maximum-entropy distributions of Fig 5A. The plot suggests that typical experimental neural recordings of 250 neurons and above are likely to lead to bimodal maximum-entropy pairwise distributions.

Under these approximations—and notwithstanding the choice of estimates that keeps the data as far away from bimodality as possible—the largest part of the data summarized by Cohen & Kohn does fall in the bimodality region of Fig 5A for *N* = 250, and almost all data lies in the bimodality region for *N* = 500; see Fig 6. These data points have only an indicative value, but suggest that our dataset is not an outlier for the bimodality problem. If those data had been recorded from a population of 500 neurons, they would have yielded a bimodal pairwise maximum-entropy model because, as shown in section “*Bimodality ranges and population size*”, the more neurons we are able to record, the more likely the bimodality occurs. Thus the bimodality problem and its consequences need to be taken seriously. Our next question is then: Is there any way to eliminate the bimodality problem?

#### Analysis of the erroneous preliminary approximation of the distribution

In the last three sections we have partly addressed the second issue raised in section “*Appearance of bimodality*”: the distribution given by the pairwise maximum-entropy method is bound to be bimodal, not only for our data, but also for typical neuronal recordings of a couple of hundreds neurons or more.

We now address the third issue: to find out more about the first erroneous approximation of the probability distribution, shown in Fig 2B. It is important to remember that it is *not* the correct pairwise maximum-entropy distribution, and the initial erroneous approximations $\hat{h}$, $\hat{J}$ of the multipliers do *not* match the data. The approximation was erroneous because the sampling phase of the Boltzmann-learning algorithm was too brief. The time required to explore the full distribution is so long that the dynamics is non-ergodic for computational purposes. This non-ergodicity effectively truncates the sampling at states ***s*** for which $\bar{s}≲\theta$, where *θ* is the population-averaged activity at the trough between the two metastable regimes. This means that if the wrong multipliers $\hat{h}$, $\hat{J}$ are used in a “truncated” distribution

$$\begin{array}{c} P_{\text{t}}\left(s|\hat{h},\hat{J},\theta \right)\propto \{\begin{array}{cc} \exp(\sum_{i}{\hat{h}}_{i}s_{i}+\sum_{i<j}{\hat{J}}_{ij}s_{i}s_{j}), & \bar{s}⩽\theta ,\\ 0, & \bar{s}>\theta , \end{array} \end{array}$$ (20)

then the expectations of this distribution are close to the experimental time averages for the single and coupled activities: E_t_(*s*_*i*_) = *m*_*i*_, E_t_(*s*_*i*_ *s*_*j*_) = *g*_*ij*_. This truncated distribution, though, is obviously *not* a pairwise maximum-entropy distribution. It is an interesting distribution nevertheless, as Fig 2B shows. Could it be obtained or approximated with the maximum-entropy method in some other way?

### Solution: An inhibited maximum-entropy model and Glauber dynamics

Let us briefly summarize our results so far and the reason why a maximum-entropy model yielding a bimodal distribution in the population-averaged activity is problematic:

- For commonly observed statistics of neuronal data, the pairwise maximum-entropy method yields a distribution with two distinct modes (bimodality), one of which at high activities—unrealistic in view of present neuroscientific data. The bimodality is bound to happen for large *N* as soon as the measured correlations are slightly positive. Moreover, the Glauber dynamics based on the pairwise model jumps between two metastable regimes and cannot be used to generate realistic surrogate data.
- The Boltzmann-learning procedure based on Glauber dynamics becomes practically non-ergodic and the Lagrange multipliers of the pairwise model are difficult or impossible to find. Standard analytic approximations fail as well.
- The initial erroneous approximation of the pairwise maximum-entropy distribution, obtained with a too short Boltzmann learning, shows an interesting fit with the data nevertheless. It would be interesting to know if it can be obtained or approximated with a generalized maximum-entropy method.

We will propose a solution that addresses all three issues at once. This solution pivots on the idea of *inhibition* and can be grasped with an intuitive explanation of how the bimodality arises.

#### Intuitive understanding of the bimodality: Mean-field picture

**Fig 7:**
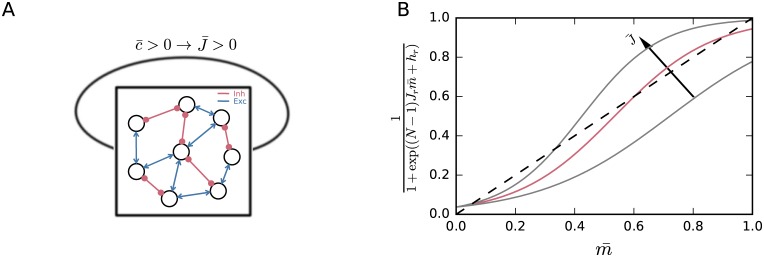
Mean-field picture. (**A**) Illustration of a self-coupled symmetric network that is self-excitatory on average. Arrow-headed blue lines (→) represent excitatory couplings; circle-headed red lines (⊸) represent inhibitory couplings. (**B**) Self-consistency solution of the naive mean-field equation, illustrated for different *J*_r_. Larger *J*_r_ lead to two additional intersections, corresponding to an unstable and a stable solution. The red curve corresponds to the *J*_r_ calculated from our experimental data Eq (16).

From the point of view of a network evolving with a Glauber dynamics with couplings ***J*** and biases ***h***, the bimodality and bistability appear because the couplings ***J*** are on average positive and make the network dominantly excitatory. The positivity of the couplings appears because the average correlation $\bar{c}$ between the neurons, empirically measured, is positive (Figs 1D and 7A). This phenomenon is not unknown: bimodalities in the distribution of extensive quantities have a similar explanation in the statistical mechanics of finite-size systems [99, 106–108].

A naive mean-field analysis also confirms this. In such an approximation we imagine that each neuron is coupled to a field representing the mean activities of all other neurons [65; 109, ch. 6]. From the point of view of entropy maximization, we are replacing the maximum-entropy distribution with one representing independent activities, having minimal Kullback-Leibler divergence from the original one [66, chs 2, 16, 17]. Given the couplings ***J*** and biases ***h***, the mean activities ***m*** must satisfy *N* self-consistency equations

$$\begin{array}{c} \frac{1}{1+\exp(\sum_{j}^{j\neq i}J_{ij}m_{j}+h_{i})}=m_{i}. \end{array}$$ (21)

In the homogeneous case they reduce to the equation ${\left\{1+\exp\left[\left(N-1\right)J_{\text{r}}\bar{m}+h_{\text{r}}\right]\right\}}^{-1}=\bar{m}$, corresponding to the intersection of two functions of $\bar{m}$: the diagonal line $\bar{m}\mapsto \bar{m}$, and the curve $\bar{m}\mapsto {\left\{1+\exp\left[\left(N-1\right)J_{\text{r}}\bar{m}+h_{\text{r}}\right]\right\}}^{-1}$ that depends parametrically on (*h*_r_, *J*_r_); see Fig 7B. For the Lagrange multipliers of our data, these curves intersect at two different values of $\bar{m}$, meaning that there are two solutions to the self-consistency equation, corresponding to two different mean activities. These approximately correspond to the maxima of the probability distribution for the population average in Fig 4A.

#### Importance of inhibition: Modified Glauber dynamics

**Fig 8:**
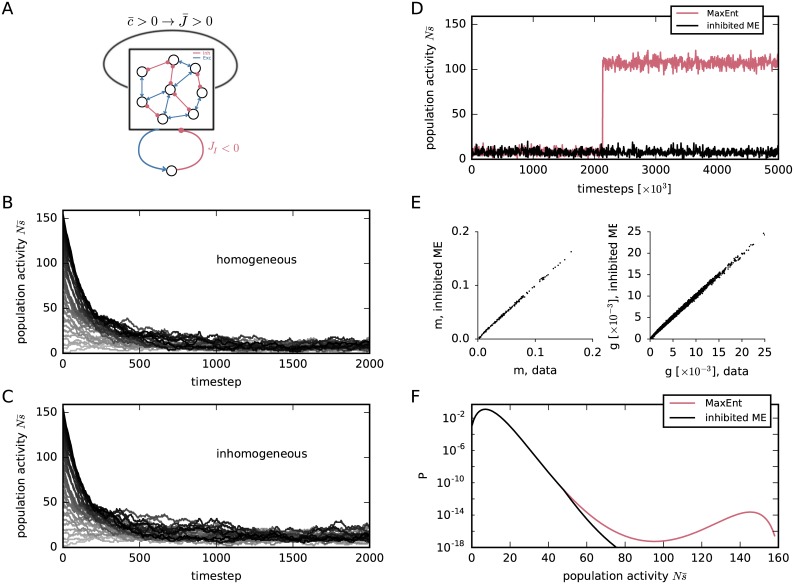
Asymmetric inhibition and elimination of bimodality and non-ergodicity. (**A**) Illustration of self-coupled network with additional asymmetric inhibitory feedback. Each neuron receives inhibitory input *J*_*I*_ < 0 from the additional neuron whenever the population-average $\bar{s}$ becomes greater than the inhibition threshold *θ*. (**B**) Population-averaged activities $\bar{s}\left(t\right)$ from several instances of the inhibited Glauber dynamics, with *J*_*I*_ = −24.7, *θ* = 0.3, and homogeneous *J*_*ij*_ = *J*_r_, *h*_*i*_ = *h*_r_ of Eq (16), as used for Fig 4B. Each instance starts with an initial population activity ***s***(0) having different values of the population average $\bar{s}\left(0\right)$, and is represented by a different grey shade, from $N\bar{s}\left(0\right)=0$ (light grey) to $N\bar{s}\left(0\right)=N$ (black). Note the disappearance, thanks to inhibition, of the bistability that was evident in the “uninhibited” case of Fig 4B. (**C**) Analogous to panel B, with *J*_*I*_ = −24.7, *θ* = 0.3, but inhomogeneous normally distributed couplings and biases as in the uninhibited case of Fig 5C. The bistability again disappears thanks to inhibition. (**D**) Comparison of a longer (5 × 10^6^ timesteps) Glauber sampling in the inhibited (black, *J*_*I*_ = −24.7, *θ* = 0.3) and uninhibited (red) case, using the couplings and biases of Fig 2D obtained from our first Boltzmann learning. (**E**) Time averages *m*_*i*_ and *g*_*ij*_ obtained from Boltzmann learning for the inhibited model *P*_i_, versus experimental ones. (**F**) Probability distribution of the population-averaged activity $P_{\text{i}}\left(\bar{s}\right)$ given by the inhibited model Eq (22) for our dataset Eq (15), compared with the one previously given by the reduced model $P_{\text{r}}\left(\bar{s}\right)$, Fig 4A.

In the neuronal network dynamics just analysed, with positive correlations on average, the second peak of high activity can be suppressed introducing an effective negative feedback loop. Such inhibitory mechanism can be represented by an additional neuronal unit *I*, having positive incoming couplings *J*_*Ik*_ > 0 and negative outgoing couplings *J*_*kI*_ < 0 with all other units *k*. This unit is therefore activated if the total activity of the other units is high, and once activated it provides negative input back to all other units. This situation is illustrated in Fig 8A. Such a stabilizing mechanism acts much in the same way as inhibition stabilizes the low-activity state in neuronal networks [110, 111]; it has also been used in the simulations by Bohte et al. [34]. The asymmetry of this mechanism is in contradiction with the symmetry of the couplings *J*_*ij*_ of the Glauber dynamics, however. We want to break this symmetry and add an asymmetric inhibitory feedback to the Glauber dynamics to avoid the bimodality of the probability distribution.

We preliminarily implement this idea in our Glauber dynamics, to observe its consequences, using first the incorrect approximations $\left(\hat{h},\hat{J}\right)$ of the multipliers that yielded a bimodal inhomogeneous pairwise distribution, and then the (correct) multipliers *h*_*i*_ = *h*_r_, *J*_*ij*_ = *J*_r_, Eq (16), of the reduced pairwise model. We connect all *N* neurons to a single inhibitory neuron that instantaneously activates whenever their average activity exceeds a threshold *θ* ∈ {0, 1/*N*, 2/*N*, …, (*N* − 1)/*N*, 1}. Upon activation the inhibitory neuron inhibits the other *N* neurons (see Fig 8A) via *N* identical negative couplings *J*_I_ < 0. The results from the simulation of the inhibited Glauber dynamics are shown in Fig 8; in all cases the inhibitory coupling was *J*_I_ = −24.7 and the inhibition threshold *θ* = 0.3.

The algorithm for sampling from this “inhibited” Glauber dynamics is explained in section “*Inhibited Glauber dynamics*”. It can be seen that the additional inhibitory neuron eliminates the bistability, leaving only the stable low-activity regime. The resulting homogeneous and inhomogeneous stationary distributions (in the inhomogeneous case *J*_*ij*_ and *h*_*i*_ are normally distributed as in Fig 5C) are either unimodal or have a second mode that is completely negligible, being tens or hundreds of orders of magnitude smaller than the first mode.

The inhibited Glauber dynamics can suppress the bistability for any network size *N*, with an appropriate choice of the inhibitory coupling *J*_I_ < 0 and threshold *θ*.

Continuing our exploration, we check what happens if we use the inhibited Glauber dynamics in the sampling phase of Boltzmann learning for our initial problem, when we tried to find a stationary distribution having means and correlations shown in Fig 1B–1D. As shown in Fig 8E, the addition of the inhibitory neuron (again with *J*_I_ = −24.7, *θ* = 0.3) eliminates the second metastable state that appeared after 2 × 10^6^ steps, cf. Fig 3. The resulting couplings and biases of the final stationary distribution are distributed as in Fig 2D—but note that this time the Boltzmann learning has converged, hence these are its correct final values.

However, it must be stressed that the stationary distribution thus found, using the inhibited Glauber dynamics, is *not* a pairwise maximum-entropy distribution, because the latter is the stationary distribution of the original Glauber dynamics, not of the modified one. If we use the inhibited dynamics in Boltzmann learning, we are abandoning the standard pairwise maximum-entropy model.

There is nevertheless a positive result: in the next section we show that the stationary distribution of the inhibited Glauber dynamics belongs to a generalized maximum-entropy family.

#### Inhibited maximum-entropy model

The pairwise maximum-entropy distribution Eq (3) is the stationary distribution of the Glauber dynamics with symmetric couplings. It is not the stationary distribution of the inhibited Glauber dynamics. But the following fact holds: *The stationary distribution of the inhibited Glauber dynamics* (Fig 8) *belongs to the maximum-entropy family*. *Its analytic expression is*

$$\begin{array}{ll} P_{\text{i}}(s|h,J,J_{\text{I}},\theta ) & =\frac{1}{Z_{\text{i}}\left(h,J,J_{\text{I}},\theta \right)}\times \\ & \exp\left[\sum_{i}h_{i}s_{i}+\sum_{i>j}J_{ij}s_{i}s_{j}+J_{\text{I}}NG\left(\bar{s}-\theta \right)\right],\\ Z_{\text{i}}\left(h,J,J_{\text{I}},\theta \right) & ≔\sum_{s}\exp\left[\sum_{i}h_{i}s_{i}+\sum_{i>j}J_{ij}s_{i}s_{j}+J_{\text{I}}NG\left(\bar{s}-\theta \right)\right],\\ G\left(\bar{s}-\theta \right) & ≔\left(\bar{s}-\theta \right)\text{H}\left(\bar{s}-\theta \right), \end{array}$$ (22)

where *J*_I_ is the (negative, in our case) coupling strength from the inhibitory neuron to the other neurons, *θ* is the activation threshold of the inhibitory neuron, and *H* is the Heaviside step function. We call Eq (22) the *inhibited pairwise maximum-entropy model*. The proof that it is the stationary distribution of the inhibited Glauber dynamics is given in section “*Inhibited Glauber dynamics*”.

**Fig 9:**
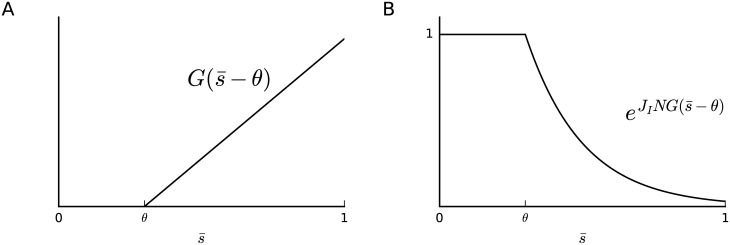
Reference measure. The function $G(\bar{s}-\theta )$ and its exponential for *J*_I_ < 0.

This maximum-entropy model is characterized by the new term $J_{\text{I}}N\;G\left(\bar{s}-\theta \right)$ in the exponential, which we call “inhibition term”. The function $G(\bar{s}-\theta )$ is plotted in Fig 9 together with its exponential. We show in section “*Expansion of the inhibition term in terms of higher-order coupled activities*” that it can also be written as a linear combination of population-averaged *K*-tuple activities, $s_{i_{1}}\;s_{i_{2}}\cdots s_{i_{K}}$, for *K* equal to *Nθ* + 1 and larger:

$$N\;G(\bar{s}-\theta )=\sum_{K=N\theta +1}^{N}\;\left(\begin{array}{c} -N\theta \\ K-N\theta -1 \end{array}\right)\;\left(\sum_{i_{1}<i_{2}<\cdots <i_{K}}s_{i_{1}}s_{i_{2}}\cdots s_{i_{K}}\right),$$ (23)

the coefficients being generalized binomial coefficients [100, ch. 6; see also 112], which have alternating signs. For example, if *N* = 5 and *θ* = 3/5,

$$N\;G(\bar{s}-\theta )=(s_{2}s_{3}s_{4}s_{5}+s_{1}s_{3}s_{4}s_{5}+s_{1}s_{2}s_{4}s_{5}+s_{1}s_{2}s_{3}s_{5}+s_{1}s_{2}s_{3}s_{4})-3s_{1}s_{2}s_{3}s_{4}s_{5}.$$ (24)

This function differs from the additional function appearing in the maximum-entropy model by Tkačik et al. [50, 94, 113], which consists in *N* + 1 constraints enforcing the observed frequency distribution of the population average $\bar{s}$. For large data samples the constraints used in those works typically equal 0 for larger values of $\bar{s}$; for reasons discussed in section “*Range of applicability of maximum-entropy models*”, the use of such extreme constraints may not be justified or meaningful.

The inhibited distribution *P*_i_(*s*) belongs to the maximum-entropy family in two different ways, the first preferable to the second:

1. It can be obtained by application of the maximum-relative-entropy (minimum-discrimination-information) principle [31, 83], with the pairwise constraints Eq (5), with respect to the reference distribution
  $$\begin{array}{c} P_{0}\left(s|J_{\text{I}},\theta \right)\propto \exp[J_{\text{I}}N\;G\left(\bar{s}-\theta \right)],\;N\theta \in \{0,1,2,\ldots ,N-1,N\}, \end{array}$$ (25)
  also called “reference measure”, which assigns decreasing probabilities to states with average activities above *θ*; see Fig 9B. This relative-maximum-entropy model can be interpreted as arising from a more detailed model in which we know that external inhibitory units make activities above the threshold *θ* increasingly improbable, like Bohte et al.’s model [34] for example. We discuss this in the “*Discussion*”. In this interpretation the parameters *J*_I_ and *θ* are chosen a priori.
2. Alternatively, it results from the application of the “bare” maximum-entropy principle given the pairwise constraints Eq (5) and an additional constraint for the expectation of $N\;G(\bar{s}-\theta )$:
  Ei(NG(s¯-θ))=∑Ns¯=NθN(Ns¯-Nθ)Pi(s¯)=∑K=NθN(-Nθ-K+1)Ei(∑i1<i2<⋯<iKsi1si2⋯siK). (26)
  This is a constraint on what could be called the “tail first moment” of the distribution for the population-averaged activity $P_{\text{i}}\left(\bar{s}\right)$: it determines whether the right tail of $P_{\text{i}}\left(\bar{s}\right)$ has a small (*J*_I_ < 0) or heavy (*J*_I_ > 0) probability. It can also be seen as a constraint on the *Nθ*-th and higher moments, owing to Eq (23). In this interpretation the parameter *J*_I_ is the Lagrange multiplier associated with this constraint, hence it is determined by the data; the parameter *θ* is chosen a priori. Note, however, that experimental data are likely to give a vanishing time average of $N\;G(\bar{s}-\theta )$, so that *J*_I_ = −∞. This interpretation has therefore to be used with care, for the reasons discussed in section “*Range of applicability of maximum-entropy models*”.

Two features of the inhibited maximum-entropy model Eq (22) are worth remarking upon:

1. The family of inhibited distributions *P*_i_ includes the pairwise family *P*_p_, Eq (3), as the particular case *J*_I_ = 0. Note that if *J*_I_ ≠ 0 the inhibited and uninhibited models with identical Lagrange multipliers (***h***, ***J***) have *different* expectations for single and coupled activities:
  $$\begin{array}{c} \begin{array}{ccc} E_{\text{i}}(s_{i}|h,J,J_{\text{I}},\theta ) & \neq & E_{\text{p}}(s_{i}|h,J),\\ E_{\text{i}}(s_{i}s_{j}|h,J,J_{\text{I}},\theta ) & \neq & E_{\text{p}}(s_{i}s_{j}|h,J), \end{array} \end{array}$$ (27)
  and therefore different covariances and correlations.
2. If the inhibitory parameter *J*_I_ is very large and negative, all activities ***s*** such that $\bar{s}>\theta$ are assigned a negligible probability by the inhibited model. We therefore have the approximation (technically, pointwise convergence as *J*_I_ → −∞)
  $$\begin{array}{c} P_{\text{i}}\left(s|h,J,J_{\text{I}},\theta \right)\propto \{\begin{array}{cc} \exp(\sum_{i}h_{i}s_{i}+\sum_{i<j}J_{ij}s_{i}s_{j}), & \bar{s}⩽\theta ,\\ 0, & \bar{s}>\theta , \end{array}\;J_{\text{I}}\ll 0, \end{array}$$ (28)
  where *C* is an appropriate normalization constant.

But the last expression is identical to Eq (20). Thus, *the inhibited maximum-entropy distribution* *P*_i_ *is approximately equal to the truncated distribution* *P*_t_—the incorrect one Eq (20) obtained with our initial Boltzmann learning—having the same multipliers (***h***, ***J***) and threshold *θ*:

$$\begin{array}{c} P_{\text{i}}\left(s|h,J,J_{\text{I}},\theta \right)\approx P_{\text{t}}\left(s|h,J,\theta \right),\;J_{\text{I}}\ll 0, \end{array}$$ (29)

and their expectations are also approximately equal. We have therefore addressed the third issue discussed in section “*Appearance of bimodality*”: the initial, incorrect but interesting approximation can actually be rescued, as we now explain.

#### Summary: Application of the inhibited maximum-relative-entropy model

Let us find a probability distribution for our data using the maximum-relative-entropy method, with reference measure Eq (25), *J*_I_ = −24.7, *θ* = 0.3, given the single and pairwise constraints Eq (5) summarized in Fig 1C and 1D. To find the Lagrange multipliers (***h***, ***J***) and the distribution Eq (22) we use the Boltzmann learning procedure with 5 × 10^6^ timesteps. For the sampling phase we must use the inhibited Glauber dynamics, because its stationary distribution is Eq (22). As proven earlier (Fig 8) no bistability arises, so we do not need to worry about wrong approximations from undersampling; in fact, a much smaller number of timesteps would suffice.

The resulting multipliers and distribution are not shown because their plots are indistinguishable to the naked eye from the homologous ones in Fig 2B–2D. We have thus found that the interesting distribution shown in red in Fig 2B, although not a pairwise maximum-entropy distribution, still belongs to an enlarged maximum-entropy family: *it is an inhibited, inhomogeneous, pairwise maximum-relative-entropy distribution*.

The inhibited maximum-relative-entropy model therefore solves all there issues presented in section “*Appearance of bimodality*”:

1. The multipliers and distribution of this model can efficiently be found via Boltzmann learning with the typical number of timesteps used in the literature (200–400 [45, 95]), because its Glauber dynamics is not affected by bistability (Fig 8).
2. This model produces a distribution that has no unrealistic modes at high activities.
3. This distribution has interesting features and fitting properties (Fig 2B).

We can ask whether the solution of these three issues by the inhibited model is enough to warrant its use. In particular, does the use of the reference measure (Eq (25)) make sense from a neurobiological standpoint? In the following “*Discussion*” we argue that it does, and that it solves in fact a fourth issue of standard (i.e. uniform-measure) maximum-entropy models for neuronal networks.

## Discussion

### Summary

In this work we have shown that pairwise maximum-entropy models, widely used as references distributions in the statistical description of the joint activity of hundreds of neurons, are poised to suffer from three interrelated problems when constrained with mean activities and pairwise correlations typically found in cortex:

1. Boltzmann-learning [64, 114] based on asynchronous Glauber dynamics [61, 63, chap. 29], used to find the Lagrange multipliers and distributions of these models, becomes practically non-ergodic (Fig 3), already for population sizes of roughly 50 neurons. The distribution is therefore difficult or impossible to find. Approximate methods like mean-field [65, 66], Thouless-Anderson-Palmer [66, 67], Sessak-Monasson [69, and refs therein] also break down in this case. This problem is known in the statistical mechanics of finite-size systems [99]. This non-ergodicity can go undetected; see “*Detection and further study of bimodality*” below.
2. Pairwise models are bound to give a *bimodal* probability distribution as soon as a critical number of units is exceeded. We have provided experimental evidence for this claim in section “*The problem: Bimodality, bistability, non-ergodicity*”. The first mode is observed in the data. But the model also predicts a second, *unobserved* mode at very high activities, with up to 90% of the population simultaneously active for long times. The probability of the second mode increases with population size. The Glauber dynamics based on this model jumps between two metastable regimes, remaining in each for long times (owing to its asynchronous update) and cannot be used to generate realistic surrogate data. As discussed in “*Is the correct pairwise model bimodal?*”, inclusion of third- or fourth-order correlations does not seem to cure this problem.
3. Interesting distributions that may be found as initial approximations of pairwise distributions (e.g. Fig 2B, red) may turn out to be incorrect owing to the first and second problems above. They have to be discarded for methodological reasons despite their interesting properties.

We have given an intuitive explanation of the common cause of these issues: positive pairwise correlations imply positive Lagrange multipliers between pairs of neuron, corresponding to a symmetric network that is excitatory on average. For typical values of correlations observed in neuroscientific experiments, this network can therefore possess two metastable dynamic regimes, given sufficiently many units. The mechanism is identical to the ferromagnetic transition in the Ising model, as explained in “*Bimodality of the inhomogeneous model for large N*”. An analogous bimodality appears in the statistical mechanics of finite-size systems [e.g. 108, 115, and refs therein]—but it is experimentally expected and verified there, unlike our neurobiological case.

Although we did not study maximum-entropy models typically used in other fields, like structural biology and genetic networks [116–118], social behavior in mammals [119, 120], natural image statistics [121, 122], and economics [123], the problems we have addressed are generic and emerge as soon as we study a large network with positive pairwise correlations on average; hence they might be of relevance to these fields.

In this work we have also suggested a remedy, based on the explanation above: the intuitive idea is to add a minimal asymmetric inhibition to the network, in the guise of an additional, asymmetrically coupled inhibitory neuron (Fig 8A) [cf. 34, p. 175]. This leads to an “inhibited” Glauber dynamics that is free from bistable regimes and has a unimodal stationary distribution *P*_i_(***s***), Eq (22). This dynamics depends on an inhibition-coupling parameter *J*_I_ and a threshold parameter *θ*.

Most important, we have shown that this new stationary distribution *P*_i_(***s***) *belongs to the maximum-entropy family*: it can be obtained with the maximum-relative-entropy method with respect to a reference measure, Eq (25) (Fig 9), that represents the neurobiologically natural presence of inhibition in the network. We call this model an “inhibited” pairwise maximum-entropy model.

The inhibited pairwise model solves all three problems above:

1. It can be found by Boltzmann learning with standard sampling times. In the present work we have not investigated whether analytic approximations like the Thouless-Anderson-Palmer or Sessak-Monasson ones can be adjusted to be applied to this model; but see point 3. below.
2. Its distribution does not have unrealistic modes at high average activities. The model allows us to decide how much any high-activity modes should be suppressed (parameter *J*_I_) and the activity above which such modes are neurobiologically unrealistic (parameter *θ*).
3. It yields distributions similar to the interesting ones that should otherwise be abandoned on methodological grounds (wrong standard pairwise distributions). In this regard, if interesting distributions found in the literature turn out to be incorrect owing to undersampling, they could be “rescued” if reinterpreted as distributions of the inhibited model; see “*Detection and further study of bimodality*” below.

### Detection and further study of bimodality

We wish to stress that the presence of bimodality and non-ergodicity can easily go unnoticed. Sampling from a bimodal distribution, the probability to switch to the second mode may be so small that it occurs over more sampling steps larger than those typically used in the literature, and the high mode is not visited during Boltzmann learning or surrogate generation. We then face a subtle situation: The obtained distribution is *not* a pairwise maximum-entropy distribution Eq (3)—the Lagrange multipliers are incorrect—yet a consistency check (also affected by undersampling) may wrongly seem to validate it, and also analytic approximations (outside of their convergence domain) may wrongly validate it.

The distribution found in this circumstance is not a standard pairwise distribution, but our *inhibited* maximum entropy distribution Eq (22), for appropriately chosen *J*_I_ and *θ*.

In this regard we urge researchers who have calculated pairwise (and even higher-order) maximum-entropy distributions for more than 50 neurons using short Boltzmann-learning procedures, to check for the possible presence of higher metastable regimes. The presence of bimodality and non-ergodicity can be checked, for example, by starting the sampling from different initial conditions, at low and high activities, looking out for bistable regimes [cf. 62, S 2.1.3]. Another way out of this problem is to use other sampling techniques or Markov chains different from the Glauber one [61, 62, 97, 98]. Alternatively, one may use the inhibited model Eq (22) with the standard approaches.

In the presence of inhomogeneous and randomly chosen parameters and large network sizes, the standard pairwise maximum-entropy distribution is mathematically identical with the Boltzmann distribution of the Sherrington & Kirkpatrick infinite-range spin glass [124, 125]. A more systematic analysis of the effect of inhomogeneity on the appearance of the second mode could therefore employ methods developed for spin glasses [126], which could produce approximate expressions for the inverse problem: the determination of Lagrange multipliers from the data. One may think of modifying the Thouless-Anderson-Palmer (TAP) mean-field approach [67, 127], generalizations of which exist for the asymmetric non-equilibrium case [93] appearing here due to the inhibitory unit. An appropriate modification of the ideas of Sessak and Monasson [68, 69] could also be an alternative. Another possibility is the use of cumulant expansions [17, 128], which unlike TAP-based approaches have the advantage of being valid also in regimes of strong coupling; recent extensions allow us to obtain the statistics at the level of individual units [129].

### Bimodality in other models

In this work we have not investigated other models, like general linear models or kinetic Ising models for example. Considering the fundamental mechanism by which the bimodality arises, we expect similar problems in other models. The reasoning backing this hypothesis is this: Pairwise correlations in cortical areas are on average positive but very weak. In this limit we expect that these correlations require slightly positive “excitatory” couplings between units in most other models; an independent-pair approximation also suggests this [127]. As a result of this rough approximation determined at the level of individual pairs, we expect the couplings to be independent of the number of units of a dynamic or statistical model. With increasing number of units in the model the overall “excitatory feedback” $\sum_{j}^{N}J_{ij}$ will increase, and a simple mean-field analysis makes us expect the appearance of a second mode at a certain critical number, what in statistical mechanics is called a ferromagnetic transition; cf. Fig 7B. We expect similar ferromagnetic transitions to happen in a wide class of statistical models that only represent the observed, on average positively correlated units. Similar transitions are also reported in Bohte et al. [34] for a biological—as opposed to statistical—neuron model composed of excitatory neurons only. In fact, they had to introduce one inhibitory neuron in their model to avoid such transitions, which is also the idea behind our inhibitory term.

The bimodality problem could be cured by allowing for asymmetric connections, enabling the implementation of possibly hidden inhibitory units that stabilize the activity. For example, kinetic Ising models [130–132], which are maximum-entropy models over the possible histories of network activity [133–135], can have positive correlations among excitatory units in the asynchronous irregular regime, while their dynamics is stabilized by inhibitory feedback [see e.g. 136, Fig 3A]. Scaling of network properties with the number of units *N* is often studied in this context. In the asynchronous regime, mean pairwise correlations decrease as *N*^−1^ [18, 22, 110, 136]. This scaling is the result of a fictive experiment, typically used to derive a theoretical results in the *N* → ∞ limit—any biological neuronal network has of course a certain fixed size *N*. The mean correlation measured in a sample of size *M*, with 1 ≪ *M* ≤ *N*, is by sampling theory expected to be roughly equal to the mean correlation of the full network, and does not vary much with *M*; only the variance around this expectation declines to 0 as *M* approaches *N*.

### Meaning and advantages of the inhibited model and its reference measure

The inhibited maximum-entropy model *P*_i_, Eq (22), solves the problems discussed above; but we may ask if this is enough to motivate its use. We consider it an interesting model for at least two reasons. First, it actually is a class of models rather than a single specific model. In the present work we have focused on its use with pairwise constraints because these are still widely discussed in the literature. But the inhibition reference measure Eq (25) can be used with higher-order constraints or other kinds of constraints as well. We leave to future works the analysis of this possibility. Second, there are neurobiological reasons why the reference measure Eq (25) can be methodologically more appropriate than the uniform measure of the standard maximum-entropy method. Let us argue this point in more depth.

Standard (i.e. uniform reference measure) maximum-entropy distributions are often recommended as “maximally noncommittal” [137]. But this adjective needs qualification. Jaynes precised: ‘“maximally noncommittal” by a certain criterion’—that the possible events or states be deemed to have a priori equal probabilities before any constraints are enforced [31]. When the initial probabilities are not deemed equal, for physical or biological reasons for example, reference measures appear. An important example of reference measure is the “density of states” that multiplies the Boltzmann factor *e*^ − *E*/(*kT*)^ in statistical mechanics [e.g. 138, ch. 16]: we cannot judge energy levels to be a priori equally probable because each one comprises a different amount of degrees of freedom. The proper choice of this reference measure is so essential as to be the first manifest difference between classical and quantum statistical mechanics, from “classical counting” to “quantum counting” of phase-space cells [138, ch. 16]. Owing to quantized energy exchanges, a quantum density of states is necessary in statistical mechanics; likewise we could say that owing to inhibitory feedback an inhibitory reference measure is necessary in the statistical mechanics of neuronal networks. The uniform reference measure of standard maximum-entropy expresses that network units have a priori equally probable {0, 1} states. But these units are neurons, whose states are not a priori equally likely. The measure of the inhibited model *P*_i_ reflects this a priori asymmetry in a simplified way. There are surely other reference measures that reflect this asymmetry in a more elaborated way, but the one we have found is likely one of the simplest; cf. Bohte et al.’s [34] inhibitory solution.

The choice of an appropriate reference measure is critically important in neuroscientific inferences also for another reason. When maximum-entropy is used to generate an initial distribution *to be updated by Bayes’s theorem*, the choice of reference measure is not critical, because a poor choice gets anyway updated and corrected as new data accumulate. Not so when maximum-entropy is used to generate a sort of reference distribution that *will not be updated*, as is often done in neuroscience: an unnaturally chosen reference measure will then bias and taint all conclusions derived from comparisons with the maximum-entropy distribution.

The inhibited pairwise model can therefore be quite useful in all applications of the maximum-entropy model mentioned in “*Introduction*”. For example, it can serve as a realistic hypothesis against which to check or measure the prominence of correlations in simulated or recorded neural activities, to separate the low baseline level of correlation from the potentially behaviourally relevant departures thereof. The surprise measure to effect such separation would, according to the inhibited model, take into account the presence of inhibition and the overall low level of activity that are natural in the cortex. The inhibited model can also be used for the generation of surrogate data which include the natural effect of inhibition besides the observed level of pairwise activity. It can also be useful in the study of the predictive sufficiency of pairwise correlations as opposed to higher-order moments, for example for distribution tails [e.g. 34–36, 38–44]; and in the characterization of dynamical regimes of neuronal activity [36, 49–51].

### Choice of inhibition parameters

The inhibition reference measure Eq (25) contains the threshold *θ* and the inhibitory coupling *J*_I_ as parameters. The choice of their values depends on the point of view adopted about the measure. Three venues seem possible: (1) One might think of choosing (*θ*, *J*_I_) to better fit the specific dataset under study, but this would counter the maximum-entropy spirit: the threshold cannot be a constraint, and the inhibitory coupling would acquire infinite values, as explained in section “*Inhibited maximum-entropy model*”. Moreover for our dataset this strategy would only give a worse fit (cf. Fig 2B) because the inhibition term flattens the distribution tails. (2) One might only want to get rid of the bistability of the Glauber dynamics and the bimodality of the distribution. In this case the precise choice of (*θ*, *J*_I_) is not critical within certain bounds. The inhibition coupling *J*_I_ < 0 must be negative and sufficiently large to suppress activity once the population-averaged activity reaches *θ*. The self-consistency condition Eq (21) then gives ${\left[1+\exp\left(\sum_{j}^{j\neq i}J_{ij}m_{j}+h_{i}+J_{\text{I}}\right)\right]}^{-1}⪡\theta$ for all *i*. The threshold *θ* can be safely set to any value between the highest observed population activity $\bar{s}$ and the second fixed point of the self-consistency equation Eq (21), which is indicative of the second mode and is beyond $\bar{s}>1/2$ (see Fig 7B) for the typically low mean activities observed in the cortex. (3) A methodologically sounder possibility, in view of the remarks about maximum-entropy measures given above, is to choose (*θ*, *J*_I_) from general neurobiological arguments and observations. This was implicitly done in Bohte & al.’s neuron model [34] for example, but unfortunately they did not publish the values they chose. We leave the discussion of the neurobiological choice of these parameters to future investigations.

### Relations to other work

Our inibition term $J_{\text{I}}N\;G\left(\bar{s}-\theta \right)$, Eq (22), formally includes Shimazaki et al.’s “simultaneous silence” constraint [44] as the limit *J*_I_ → −∞, *θ* = 1/*N*. Because of this limit their model has a sharp jump in probability at $\bar{s}=1/N$: their constraint uniformly removes probability for $\bar{s}>1/N$ and assigns it to the single point $\bar{s}=0$. In contrast, our inhibited model *P*_i_ presents a kink but no jump for $\bar{s}=\theta$, with a discontinuity in the derivative proportional to *J*_I_. But besides this mathematical relationship, our inhibition term and the “simultaneous silence” constraint have different motivations and uses. As discussed at length above and in section “*Inhibited maximum-entropy model*”, our term is best interpreted as a reference measure expressing the effects of inhibition, providing a biologically more suitable starting point [cf. 34] for maximum-entropy, rather than a constraint. Its goal is not to improve the goodness-of-fit for activities well below threshold, in contrast to earlier works [e.g. 35, 40, 50, 78, 80] and to the “simultaneous silence” constraint [44]. The goodness-of-fit is determined by the constraints alone. In this regard we do not present any improvement of the fit compared to a pure pairwise model. Future work could explore combinations of the here proposed reference measure and additional constraints that improve the fitness of the model.

## Materials and methods

### Range of applicability of maximum-entropy models

Maximum-entropy models are an approximate limit case of probability models by *exchangeability* [139–141], or *sufficiency* [141, 142, §§4.2–5]. This approximation holds if the constraints are empirical averages (e.g. time averages in our case) over enough many data compared with the number of points in the sample space. How much is “enough” depends on where the empirical averages lie within their physically allowed ranges: If they are well within their ranges, then a number of data values large but still smaller than the number of sample-space points may be enough. If the empirical averages are close or equal to their physically allowed extreme values, then the number of data values should be much larger than the number of sample-space points. If these conditions are not met the maximum-entropy method gives unreasonable or plainly wrong results, as can be ascertained by comparison with the non-approximated Bayesian model. Simple examples of these limitations are illustrated in [140, 141] together with the more reasonable predictions of the non-approximated Bayesian models [see also 61, p. 308].

A very large positive or negative Lagrange multiplier usually signals that the maximum-entropy method is inadequate, because the constraint corresponding to the multiplier is approaching its minimal or maximal allowed values. Consider our case, discussed in section “*The problem: Bimodality, bistability, non-ergodicity*”. The constraints are time-averages over roughly 300000 data points, and the sample space—the possible network states—has 2^*N*^ = 2^159^ ≈ 7 × 10^47^ points. Suppose that we want to use as constraints the *N* + 1 observed frequencies of the total activity $N\bar{s}$ [cf. 50, 94, 113]. Each frequency is bounded between 0 and 1. In our data the values $N\bar{s}=24$ and $N\bar{s}=28$ have non-zero frequencies, but the intermediate values $N\bar{s}\in \left\{25,26,27\right\}$ have zero frequencies—the minimum possible value. The Lagrange multipliers for the latter three frequencies would be −∞. The maximum-entropy model would therefore predict that *it is possible for the network to have 24 or 28 simultaneuosly active neurons, but impossible for it to have 25, 26, or 27 active neurons*–not even in future recordings, if we interpret the model that way. Such a prediction is unreasonable, not to say a little silly. Under the assumption that each neuron is as likely as not to be active in each time bin, the probability that in 300000 time bins we observe all possible values of the total activity $N\bar{s}\in \left\{0,\ldots ,159\right\}$—each at least once—is of the order 10^−1463^. This means that it is practically certain that some values of $N\bar{s}$ will not appear in our recording; not because of physical impossibility, but because of the exceedingly small number of observations compared with that of possible events. It is unreasonable to think that the three values 25, 26, 27 could not appear in a longer recording, yet the values 24 and 28 could. As signalled by the large value of the Lagrange multipliers, the conditions for the validity of the maximum-entropy limit are not satisfied in this case, and the method breaks down. The validity of the inhomogeneous pairwise model is similarly questionable if there are neuron pairs with zero coupled activity, *g*_*ij*_ = 0; some corrections to the method are necessary in that case.

The limitations of the maximum-entropy method are well-known [143] in the field of image reconstruction of astronomical sources, where this method has probably most successfully been applied for the longest time. In this field the maximum-entropy principle is today used differently: to generate a distribution on the space of prior distributions, rather than a prior itself [144, 145].

### Glauber dynamics

Here we show that there is a temporal process that is able to sample from the the distribution *P*_p_(***s***|***h***, ***J***) Eq (3). This temporal dynamics is called *Glauber dynamics*. It is an example of a Markov chain on the space of binary neurons {0, 1}^*N*^ [63]. At each time step a neuron *s*_*i*_ is chosen randomly and updated with the update rule

$$\begin{array}{ccc} s_{i} & \leftarrow & 1\;\text{with}\;\text{probability}\;F_{i}\left(s\right)=g(\sum_{k}^{k\neq i}J_{ik}s_{k}+h_{i})\;\text{and}\;0\;\text{else} \end{array}$$ (30)

$$\begin{array}{ccc} g(x) & = & \frac{1}{1+\exp(-x)}, \end{array}$$ (31)

where the coupling is assumed to be symmetric, *J*_*ij*_ = *J*_*ji*_, and self-coupling is absent, *J*_*ii*_ = 0. The transition operator of the Markov chain, *κ*, only connects states that differ by at most one neuron, so for the transition of neuron *i* we can write, if $s^{i+}=\left(s_{1},\ldots ,\underset{i-th}{\underset{︸}{1}},\ldots ,s_{N}\right)$ and $s^{i-}=\left(s_{1},\ldots ,\underset{i-th}{\underset{︸}{0}},\ldots ,s_{N}\right)$,

$$\begin{array}{ccc} \kappa (s^{i+}|s^{i-}) & = & F_{i}\left(s^{i-}\right)\\ \kappa (s^{i-}|s^{i+}) & = & 1-F_{i}\left(s^{i+}\right). \end{array}$$ (32)

The pairwise maximum-entropy distribution *P*_p_(***s***|***h***, ***J***) is stationary under the Markov dynamics above. The proof can be obtained as the *J*_I_ = 0 case of the proof, given below, for the inhibited pairwise maximum-entropy model.

### Inhibited Glauber dynamics and its stationary maximum-entropy distribution

#### Inhibited Glauber dynamics

In the “inhibited” Glauber dynamics, the network of *N* neurons with states *s*_*i*_(*t*) has an additional neuron with state *s*_I_(*t*). The dynamics is determined by the following algorithm starting at time step *t* with states ***s*** = ***s***(*t*), *s*_I_ = *s*_I_(*t*):

1. One of the *N* units is chosen, each unit having probability 1/*N* of being the chosen one. Suppose *i* is the selected unit.
2. The chosen unit *i* is updated to the state $s_{i}^{\prime }≔s_{i}\left(t+1\right)$ with probability
  $$\begin{array}{c} \begin{array}{cc} p(s_{i}^{\prime }|s,s_{\text{I}}) & ={\left(1+\exp[\left(1-2s_{i}^{\prime }\right)F_{i}\left(s,s_{\text{I}}\right)]\right)}^{-1}\\ & =\{\begin{array}{cc} {\left[1+e^{F_{i}\left(s,s_{\text{I}}\right)}\right]}^{-1}, & \text{for}\;s_{i}^{\prime }=0,\\ {\left[1+e^{-F_{i}\left(s,s_{\text{I}}\right)}\right]}^{-1}, & \text{for}\;s_{i}^{\prime }=1, \end{array} \end{array}\\ \text{with}\;F_{i}\left(s,s_{\text{I}}\right)≔h_{i}+\sum_{k}^{k\neq i}J_{ik}s_{k}+J_{\text{I}}s_{\text{I}}. \end{array}$$
  Note the additional coupling from the neuron *s*_I_, with strength *J*_I_. This strength can have any sign, but we are interested in the *J*_I_ ⩽ 0 case; we therefore call *s*_I_ the “inhibitory neuron”.
3. The inhibitory neuron is deterministically updated to the state $s_{\text{I}}^{\prime }≔s_{\text{I}}\left(t+1\right)$ given by
  $$\begin{array}{c} s_{\text{I}}^{\prime }=H(\sum_{k}s_{k}/N-\theta ), \end{array}$$ (33)
  corresponding to a Kronecker-delta conditional probability
  $$\begin{array}{c} p\left(s_{\text{I}}^{\prime }|s,s_{\text{I}}\right)=p\left(s_{\text{I}}^{\prime }|s\right)=\delta [s_{\text{I}}^{\prime }-H(\sum_{k}s_{k}/N-\theta )]. \end{array}$$ (34)
  In other words, the inhibitory neuron becomes active if the population-averaged activity of the other neurons is equal to or exceeds the threshold *θ*.
4. The time is stepped forward, *t* + 1 → *t*, and the process repeats from step 1.

The original Glauber dynamics, described in the previous section, is recovered when *J*_I_ = 0, which corresponds to decoupling the inhibitory neuron *s*_I_.

The total transition probability can be written as

$$p\left(s^{\prime },s_{\text{I}}^{\prime }|s,s_{\text{I}}\right)=\frac{1}{N}\;\delta \;[s_{\text{I}}^{\prime }-H(\sum_{k}s_{k}/N-\theta )]\times \sum_{i}[{\left(1+\exp[\left(1-2s_{i}^{\prime }\right)F_{i}\left(s,s_{\text{I}}\right)]\right)}^{-1}\;\prod_{k}^{k\neq i}\delta \left(s_{k}^{\prime }-s_{k}\right)];$$ (35)

the product of Kronecker deltas in the last term ensures that at most one of the *N* neurons changes state at each timestep.

The transition probabilities for the chosen neuron *s*_*i*_ and the inhibitory neuron *s*_I_ are independent, conditional on the state of the network at the previous timestep:

$$\begin{array}{c} p\left(s^{\prime },s_{\text{I}}^{\prime }|s,s_{\text{I}}\right)=p\left(s^{\prime }|s\right)\;p\left(s_{\text{I}}^{\prime }|s\right), \end{array}$$

so the transition probability for the *N* neurons only can be written as

$$\begin{array}{c} p\left(s^{\prime }|s\right)=\frac{1}{N}\;\sum_{i}[{\left(1+\exp[\left(1-2s_{i}^{\prime }\right)F_{i}\left(s\right)]\right)}^{-1}\;\prod_{k}^{k\neq i}\delta \left(s_{k}^{\prime }-s_{k}\right)], \end{array}$$ (36)

$$\begin{array}{c} \text{with}\;F_{i}\left(s\right)≔h_{i}+\sum_{k}^{k\neq i}J_{ik}s_{k}+J_{\text{I}}\;H(\sum_{k}s_{k}/N-\theta )]. \end{array}$$ (37)

This formula also shows that the transition probability for the network can alternatively be derived without explicitly introducing an inhibitory unit: starting from the modified activation function

$$p(s_{i}|s)={\left\{1+\exp\left[\left(1-2s_{i}\right)\;F_{i}\left(s\right)\right]\right\}}^{-1}$$

with *F*_*i*_ defined by Eq (39), the transition probability Eq (36) for the network follows from the additional requirement that only a single unit changes state within a single update.

#### Proof that the inhibited maximum-entropy model is the stationary distribution of the inhibited Glauber dynamics

The modified maximum-entropy distribution *P*_i_, Eq (22), is the stationary distribution of a slightly modified version of the above dynamics, with the update rule

$$\begin{array}{c} s_{\text{I}}^{\prime }=H(\sum_{k}^{k\neq i}s_{k}/N-\theta ), \end{array}$$ (38)

and the use of *N* inhibitory neurons, one for each of the original *N* units. This dynamics has a slightly different transition probability, with activation function

$$\begin{array}{c} F_{i}\left(s\right)≔h_{i}+\sum_{k}^{k\neq i}J_{ik}s_{k}+J_{\text{I}}\;H(\sum_{k}^{k\neq i}s_{k}/N-\theta )] \end{array}$$ (39)

instead of Eq (37). Note that the two dynamics are very similar for large enough *N*. To prove the stationarity of inhibited maximum-entropy distribution *P*_i_, we show that *P*_i_ satisfies the detailed-balance equality

$$\begin{array}{c} p\left(s^{\prime }|s\right)\;P_{\text{i}}\left(s\right)=p\left(s|s^{\prime }\right)\;P_{\text{i}}\left(s^{\prime }\right)\;\text{or}\;\frac{p(s^{\prime }|s)}{p(s|s^{\prime })}=\frac{P_{\text{i}}\left(s^{\prime }\right)}{P_{\text{i}}\left(s\right)},\;\forall s,s^{\prime }, \end{array}$$ (40)

which is a sufficient condition for stationarity [146–148].

First note that if ***s***′ and ***s*** differ in the state of more than one neuron, the transition probability *p*(***s***′|***s***) vanishes and the detailed-balance above is trivially satisfied. Also the case ***s***′ = ***s*** is trivially satisfied. Only the case in which ***s***′ and ***s*** differ in the state of one unit, say *s*_*i*_, remains to be proven. Assume then that

$$\begin{array}{c} s_{i}^{\prime }=1,\;s_{i}=0,\;\forall k\neq i,\;s_{k}^{\prime }=s_{k}; \end{array}$$ (41)

by symmetry, if the detailed balance is satisfied in the case above it will also be satisfied with the values 0 and 1 interchanged.

Substituting the transition probability Eqs (36) and (39) in the left-hand side of the fraction form of the detailed balance Eq (40), and noting that *F*_*i*_(***s***′) = *F*_*i*_(***s***), we have

$$\begin{array}{c} \begin{array}{cc} \frac{p(s^{\prime }|s)}{p(s|s^{\prime })} & =\exp{\left[-F_{i}\left(s\right)\right]}^{-1}\\ & =\exp[h_{i}+\sum_{k}^{k\neq i}J_{ik}s_{k}+J_{\text{I}}\;H(\sum_{k}^{k\neq i}s_{k}/N-\theta )]. \end{array} \end{array}$$ (42)

Using the expression for the inhibited model *P*_i_, Eq (22), in the right-hand side of the fraction form of the detailed balance Eq (40), we have

$$\begin{array}{c} \begin{array}{cc} \frac{P(s^{\prime })}{P(s)} & =\exp\{[\sum_{k\neq i}h_{k}s_{k}+h_{i}\cdot 1\\ & \;\;+\frac{1}{2}\sum_{k,m\neq i}^{k\neq m}J_{mk}s_{m}s_{k}+\sum_{k\neq i}J_{mi}s_{m}\cdot 1\\ & \;\;+J_{\text{I}}N\;G(\sum_{k}^{k\neq i}\frac{s_{k}}{N}+\frac{1}{N}-\theta )]\\ & \;-[\sum_{k\neq i}h_{k}s_{k}+h_{i}\cdot 0\\ & \;\;+\frac{1}{2}\sum_{k,m\neq i}^{k\neq m}J_{mk}s_{m}s_{k}+\sum_{k\neq i}J_{mi}s_{m}\cdot 0\\ & \;\;+J_{\text{I}}N\;G(\sum_{k}^{k\neq i}\frac{s_{k}}{N}+\frac{0}{N}-\theta )]\}\\ & =\exp[h_{i}+\sum_{k}^{k\neq i}J_{ik}s_{k}+J_{\text{I}}\;H(\sum_{k}^{k\neq i}s_{k}/N-\theta )], \end{array} \end{array}$$ (43)

where we have used the equality *NG*(*x* + 1/*N*) − *NG*(*x*) = *H*(*x*), valid if $x=\sum_{k}^{k\neq i}s_{k}/N-\theta$ and *Nθ* ∈ **Z**. Comparison of formulae Eqs (42) and (43) finally proves that the detailed balance is satisfied also in the case Eq (41).

### Simulation of Glauber dynamics with NEST

The neuron model ginzburg_neuron in NEST, a simulator for neural network models [96], implements the Glauber dynamics, if the parameters of the gain function are chosen appropriately. The gain function has the form

$$\begin{array}{c} g_{\text{ginzburg}}\left(h\right)=c_{1}h+\frac{c_{2}}{2}(1+\tanh\left(c_{3}\left(h-\theta \right)\right). \end{array}$$ (44)

With $\tanh\left(x\right)=\frac{e^{x}-e^{-x}}{e^{x}+e^{-x}}$, setting *x* = *c*_3_(*h*− *θ*), *c*_1_ = 0, *c*_2_ = 1, $c_{3}=\frac{1}{2}$ it takes the form

$$\begin{array}{c} \begin{array}{cc} g_{\text{ginzburg}}\left(h\right) & =\frac{1}{2}\frac{e^{x}+e^{-x}+e^{x}-e^{-x}}{e^{x}+e^{-x}},\\ & =\frac{1}{1+e^{-2x}}=\frac{1}{1+e^{-(h-\theta )}}, \end{array} \end{array}$$ (45)

which is identical to Eq (31).

### Bimodality of the inhomogeneous model for large *N*

The large *N* limit for the inhomogeneous pairwise model can be studied employing results from spin glass theory [125]. The first point to realize is that for weak correlations the Lagrange multipliers *J*_*ij*_ are to dominant order determined only by the covariances between units *i* and *j* and by their respective mean activities. This follows from eq. (7) of Roudi et al. 2009 [127], which we expand in the limit of weak correlations (and hence only to linear order in *J*_*ij*_) as

$$\begin{array}{c} -J_{ij}+O\left(J_{ij}^{2}\right)\overset{\text{Roudi}\;\text{et}\;\text{al}.\;\text{eq}.\;7}{\overset{↓}{=}}{\left[C^{-1}\right]}_{i\neq j}={\left[\left\{m_{k}\left(1-m_{k}\right)\;\delta_{kl}+c_{k\neq l}\right\}\right]}_{ij}^{-1}\\ ={\left[{\left\{m_{k}\left(1-m_{k}\right)\delta_{kl}\right\}}^{-\frac{1}{2}}{\left(\left\{\delta_{kl}+\frac{c_{k\neq l}}{\sqrt{m_{k}\left(1-m_{k}\right)\;m_{l}\left(1-m_{l}\right)}}\right\}\right)}_{ij}^{-1}{\left\{m_{l}\left(1-m_{l}\right)\delta_{kl}\right\}}^{-\frac{1}{2}}\right]}_{i\neq j}\\ ≃-\frac{c_{ij}}{m_{k}\left(1-m_{k}\right)\;m_{l}\left(1-m_{l}\right)}+O\left(c_{ij}^{2}\right), \end{array}$$

where we used the geometric series from the second to the third line. Since considering larger networks will not change the statistics of the *c*_*ij*_ (as long as we are within the local network of *N* ≃ 10^3^–10^4^ neurons), the Lagrange multipliers *J*_*ij*_ will, to leading order, follow the same distribution. In particular their population mean ${\bar{J}}_{ij}=\frac{1}{N(N-1)}\sum_{ij}J_{ij}\overset{N\gg 1}{\to }\mu$ and their variance $\bar{\delta J_{ij}^{2}}=\frac{1}{N(N-1)}\sum_{ij}{\left(J_{ij}-{\bar{J}}_{ij}\right)}^{2}\overset{N\gg 1}{\to }\sigma^{2}$ converge to values *μ* and *σ*^2^ that are, to leading order, independent of *N*.

We now consider the “energy” associated with the maximum-entropy model

$$\begin{array}{cc} E(s) & =-\frac{1}{2}\sum_{ij}\;J_{ij}s_{i}s_{j}-\sum_{i}\;h_{i}s_{i}. \end{array}$$

For this expression to possess a well-defined *N* → ∞ limit, we need (see [125], eqs. 1.3a and 1.3b) that $\mu ={\tilde{J}}_{0}/N$ and $\sigma^{2}={\tilde{J}}^{2}/N$, with *N*-independent quantities denoted by a tilde. We may therefore determine at which point we are in the phase diagram, shown in Fig 1 of [125]. So we obtain the scaling relations

$$\begin{array}{cc} {\tilde{J}}_{0} & =N\;\mu . \end{array}$$

$$\begin{array}{cc} \tilde{J} & =\sqrt{N}\;\sigma . \end{array}$$

We may now study what happens if we increase *N*. We therefore investigate how, for given and *N*-independent values of *μ* and *σ*, we move through the phase diagram of the model (see Fig 1 in [125]). The axes of this diagram are spanned by

$$\begin{array}{cc} \frac{{\tilde{J}}_{0}}{\tilde{J}} & =\sqrt{N}\;\frac{\mu }{\sigma }, \end{array}$$

$$\begin{array}{cc} \frac{kT}{\tilde{J}} & =\frac{1}{\sqrt{N}\;\sigma }. \end{array}$$

So increasing *N* we will move to the lower right in the phase diagram, ultimately crossing the transition to ferromagnetic behaviour. This is the point at which the model becomes bistable. One may note that the position of this cross-over is not entirely correctly predicted by the replica-symmetric theory of [125]. The true solution, found by Parisi [149] is slightly displaced compared to the transition line in the diagram in Fig 1 of [125]. Still, as we are only interested in the limit *N* → ∞, the result is the same and the model becomes bistable.

### Expansion of the inhibition term in terms of higher-order coupled activities

Higer-order correlations are represented by products of *K* distinct activities, like e.g. *s*_1_ *s*_3_ *s*_4_ *s*_9_, with *K* ∈ {0 …, *N*}, whose expectations are the raw *K*-th moments of the distribution. There are (NK) such products for each given *K*. For a network activity (*s*_1_, ⋯, *s*_*N*_) ∈ {0, 1}^*N*^, each of those products amounts to either 0 or 1. More precisely, if the total activity is *S*, then (SK) of these products will equal 1 and the others will vanish; the binomial vanishes by definition if *K* > *S*, so it covers this case as well.

In the reduced, homogeneous case we can meaningfully sum together all products with *K* factors, because they have the same probability. Then, from what we said above, such sum equals (SK) when the total activity is *S*:

∑i1<i2<⋯<iKsi1si2⋯siK=(SK)if∑isi=S. (46)

We want to rewrite the logarithm of the inhibition term $NG\left(\bar{s}-\theta \right)≔N\;\left(\bar{s}-\theta \right)\;H\left(\bar{s}-\theta \right)$ as a sum of such sums of *K* products, in order to interpret it as a combination of higher-order correlations:

NG(s¯-θ)=∑K=0NfK(∑i1<i2<⋯<iKsi1si2⋯siK)=∑K=0NfK(Ns¯K), (47)

with *θ*-dependent coefficients *f*_*K*_. Let us find them.

Rewrite $NG(\bar{s}-\theta )$ as *G*(*S* − *Θ*), with $S≔N\bar{s}\in \left\{0,\ldots ,N\right\}$ and *Θ* ≔ *Nθ*. The (*N* + 1)-tuple *v* of numbers

$$\begin{array}{c} v_{\cdot }≔(G(0-\Theta ),G(1-\Theta ),\ldots ,G(N-\Theta ))=\left(0,\ldots ,\underset{\Theta \text{th}}{\underset{︸}{0}},1,\ldots ,N-\Theta \right) \end{array}$$

is a *θ*-dependent row-vector.

Expression Eq (47) can be interpreted as the matrix product *fP* of the row vector *f*–which we want to find—by the (*N* + 1)-dimensional matrix *P* having element (SK) in its (*K* + 1)th row and (*S* + 1)th column. Such matrix is called a *Pascal* matrix [150, 151]; for example, for *N* = 4,

$$\begin{array}{c} {\left(P\right)}_{K}^{S}=(\begin{array}{ccccc} 1 & 1 & 1 & 1 & 1\\ 0 & 1 & 2 & 3 & 4\\ 0 & 0 & 1 & 3 & 6\\ 0 & 0 & 0 & 1 & 4\\ 0 & 0 & 0 & 0 & 1 \end{array}). \end{array}$$

Hence we have *v* = *fP*, and therefore *f* = *vP*^−1^. The inverse *P*^−1^ of a Pascal matrix has elements (P-1)SK=(-1)K-S(KS) [150, 151]; for example, for *N* = 4,

$$\begin{array}{c} {\left(P^{-1}\right)}_{S}^{K}=(\begin{array}{ccccc} 1 & -1 & 1 & -1 & 1\\ 0 & 1 & -2 & 3 & -4\\ 0 & 0 & 1 & -3 & 6\\ 0 & 0 & 0 & 1 & -4\\ 0 & 0 & 0 & 0 & 1 \end{array}). \end{array}$$

By multipling the expressions of *v* and *P*^−1^ above we find that the row-vector *f* = *vP*^−1^ is, explicitating its dependence on *Θ*,

$$\begin{array}{c} f_{K}\left(\Theta \right)=\{\begin{array}{ccccccccccc} (0 & 1 & 0 & 0 & 0 & 0 & 0 & \cdots & & & \Theta =0\\ (0 & 0 & 1 & -1 & 1 & -1 & 1 & \cdots & & & \Theta =1\\ (0 & 0 & 0 & 1 & -2 & 3 & -4 & \cdots & & & \Theta =2\\ (0 & 0 & 0 & 0 & 1 & -3 & 6 & \cdots & & & \cdots \\ (0 & 0 & 0 & 0 & 0 & 1 & -4 & \cdots \\ \cdots \\ K=0 & K=1 & K=2 & \cdots \end{array}. \end{array}$$

This solution has a convenient feature: if we increase *N* by 1, the matrix (*f*_*K*_(*Θ*)) of the *N*-dimensional solution acquires one new row and column, but the already existing entries remain unchanged.

We can thus write *G*(*S* − *Θ*) as ∑K=0NfK(Θ)(SK), with *f*_*K*_(*Θ*) given above. But *f*_*K*_(*Θ*) = 0 if *K* ⩽ *Θ*, so we can also restrict the sum to *K* > *Θ*:

G(S-Θ)=∑K=Θ+1NfK(Θ)(SK). (48)

Compare the matrix of values for *f*_*K*_(*Θ*) above with that for the generalized binomial coefficient [100, 112]:

(−ΘK−Θ−1)=(-1)K-Θ-1(K-2K-Θ-1)={100000⋯Θ=01-11-11⋯Θ=11-23-4⋯Θ=21-36⋯⋯1-4⋯⋯K=1K=2⋯;

if *K* > *Θ* we have $f_{K}\left(\Theta \right)=\left(\begin{array}{c} -\Theta \\ K-\Theta -1 \end{array}\right)$. We can therefore write Eq (48) more explicitly, recalling that $S\equiv N\bar{s}$, *Θ* ≡ *Nθ*, $G\left(S-\Theta \right)\equiv NG\left(\bar{s}-\theta \right)$, and Eq (46), as:

NG(s¯-θ)=∑K=Nθ+1N(-NθK-Nθ-1)(Ns¯K),≡∑K=Nθ+1N(-NθK-Nθ-1)(∑i1<i2<⋯<iKsi1si2⋯siK),

which is formula Eq (23).
